# Supplementary material for: IL-26 from innate lymphoid cells regulates early-life gut epithelial homeostasis by shaping microbiota composition
Source: EMBO J. 2025 Oct 22;44(23):6832–56. doi: 10.1038/s44318-025-00588-w (PMC12669248; doi:10.1038/s44318-025-00588-w)
Supplement: Supplementary file 1 — Appendix [file 44318_2025_588_MOESM1_ESM.pdf]

Table of content

Appendix Figure S1. *il26* is expressed in the zebrafish larval gut……………………………2

Appendix Figure S2. Generation of *il26*-defecient zebrafish………………………..…………3

Appendix Figure S3. rzIl26 administration does not influence proliferation and DNA damage in the larval gut……………………………………………………………………………..……4

Appendix Figure S4. *il26*$^{-/-}$ adult fish do not display overt phenotypes…………………………...5

Appendix Figure S5. Generation of *il20ra*-defecient zebrafish……………………..…………6

Appendix Figure S6. Immunostaining in WT and *il26*$^{-/-}$ larval guts reared CV, GF, or cohoused...7

Appendix Figure S7. Immunostaining in WT and *il26*$^{-/-}$ larval guts upon *E. tarda* infection……8

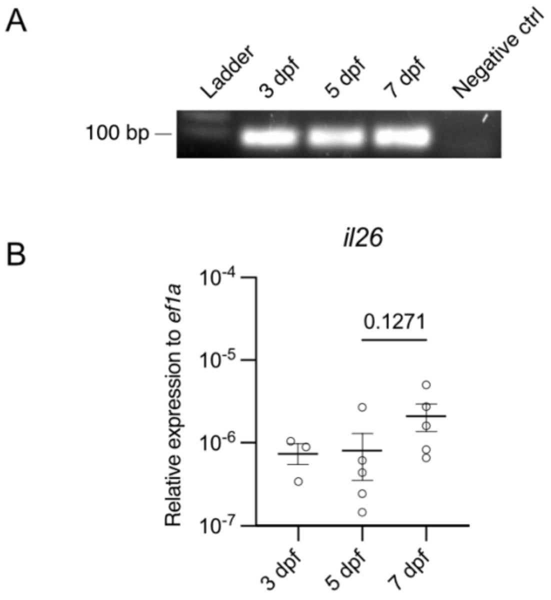

Appendix Figure S1. *il26* is expressed in the zebrafish larval gut. (**A**) RT-PCR analysis of *il26* expression in zebrafish larvae at 3, 5, and 7 dpf. A PCR product of the expected size (~100 bp) was detected at all time points. (**B**) qRT-PCR analysis of *il26* in dissected guts of WT at 3, 5, and 7 dpf. Data information: (**B**) Data are presented as mean ± SEM. Sample sizes were as follows (**B**: $n_{3dpf} = 3$, $n_{5dpf} = 5$, $n_{7dpf} = 5$). n represents the number of biological replicates (minimum of 3 independent experiments). Statistical significance was determined by Kruskal-Wallis test (**B**). Source data are available online for this figure.

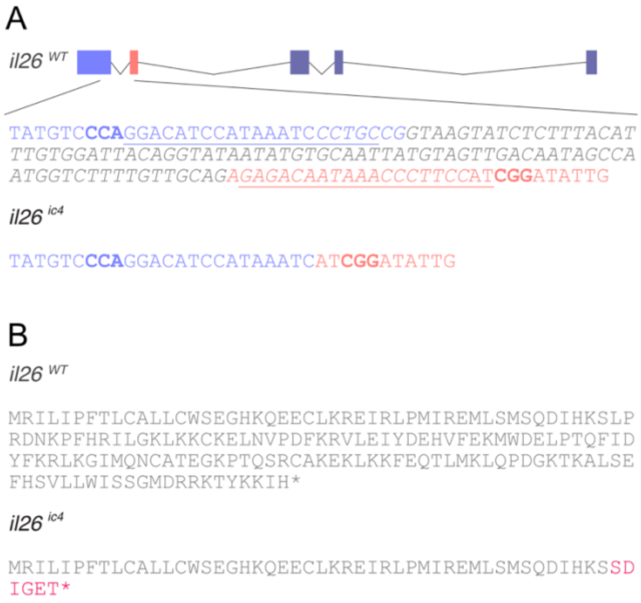

Appendix Figure S2. Generation of *il26*-defecient zebrafish. (**A**) Schematics and DNA sequence of the WT and mutant zebrafish *il26* alleles. The single guide RNA (sgRNA) target sites are underlined. The protospacer adjacent motif (PAM) sequences are indicated in bold. The deleted nucleotides in *il26*^{ic4} are highlighted in *italics*. Our approach resulted in a 110-bp deletion across exon 1 and exon 2 of the zebrafish IL-26 gene (*il26*^{ic4}). (**B**) The predicted protein sequence of *il26*^{ic4} consisting of 53-amino acids.

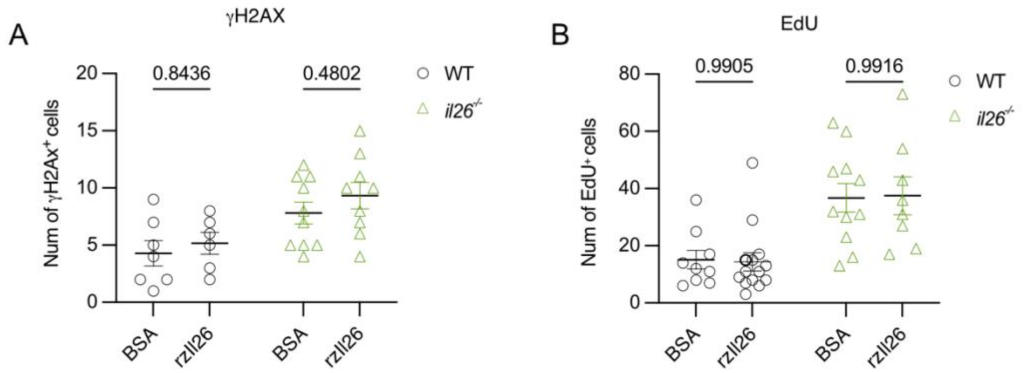

Appendix Figure S3. rzIl26 administration does not influence proliferation and DNA damage in the larval gut. (**A**, **B**) Quantification of γH2AX (**A**) and EdU (**B**) staining in WT and *il26*$^{-/-}$ posterior larval guts following rzIl26 administration. Data information: (**A**, **B**) Data are presented as mean ± SEM. Sample sizes were as follows (**A**: $n_{WT\ BSA}$ = 7, $n_{WT\ rzIl26}$ = 6, $n_{il26-/-\ BSA}$ = 10, $n_{il26-/-\ rzIl26}$ = 9; **B**: $n_{WT\ BSA}$ = 9, $n_{WT\ rzIl26}$ = 14, $n_{il26-/-\ BSA}$ = 11, $n_{il26-/-\ rzIl26}$ = 8). n represents the number of biological replicates (minimum of 3 independent experiments). Statistical significance was determined by 2way ANOVA (**A**, **B**). Source data are available online for this figure.

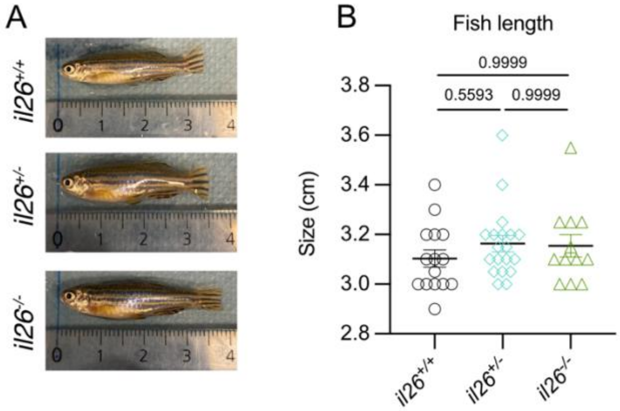

Appendix Figure S4. *il26$^{-/-}$* adult fish do not display overt phenotypes. (**A**, **B**) Images of *il26$^{+/+}$*, *il26$^{+/-}$*, and *il26$^{-/-}$* adult fish (**A**) and their length (**B**). Data information: (**B**) Data are presented as mean ± SEM. Sample sizes were as follows (**B**: $n_{il26+/+} = 15$, $n_{il26+/-} = 19$, $n_{il26-/-} = 12$). n represents the number of biological replicates (minimum of 3 independent experiments). Statistical significance was determined by Kruskal-Wallis test (**B**). Source data are available online for this figure.

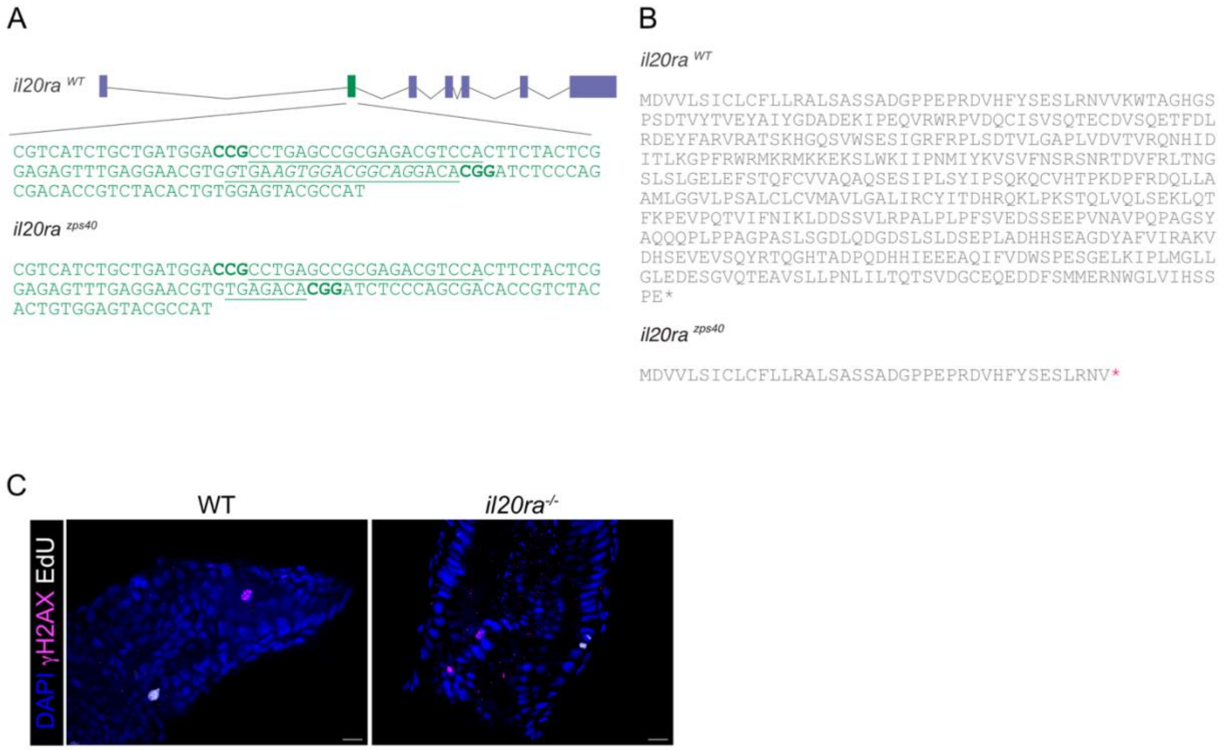

Appendix Figure S5. Generation of *il20ra*-defecient zebrafish. (**A**) Schematics and DNA sequence of the WT and mutant zebrafish *il20ra* alleles. The single guide RNA (sgRNA) target sites are underlined. The protospacer adjacent motif (PAM) sequences are indicated in bold. The deleted nucleotides in *il20ra*^{zps40} are highlighted in *italics*. This led to a deletion in the 2nd exon of the zebrafish *il20ra* gene (*il20ra*^{aps40}). (**B**) The predicted protein sequence for *il20ra*^{zps40} consisting of 40 amino acids. (**C**) Representative images of EdU and γH2AX staining in WT and *il20ra*$^{-/-}$ 5-dpf larval guts. Scale bars: 10 µm. Source data are available online for this figure.

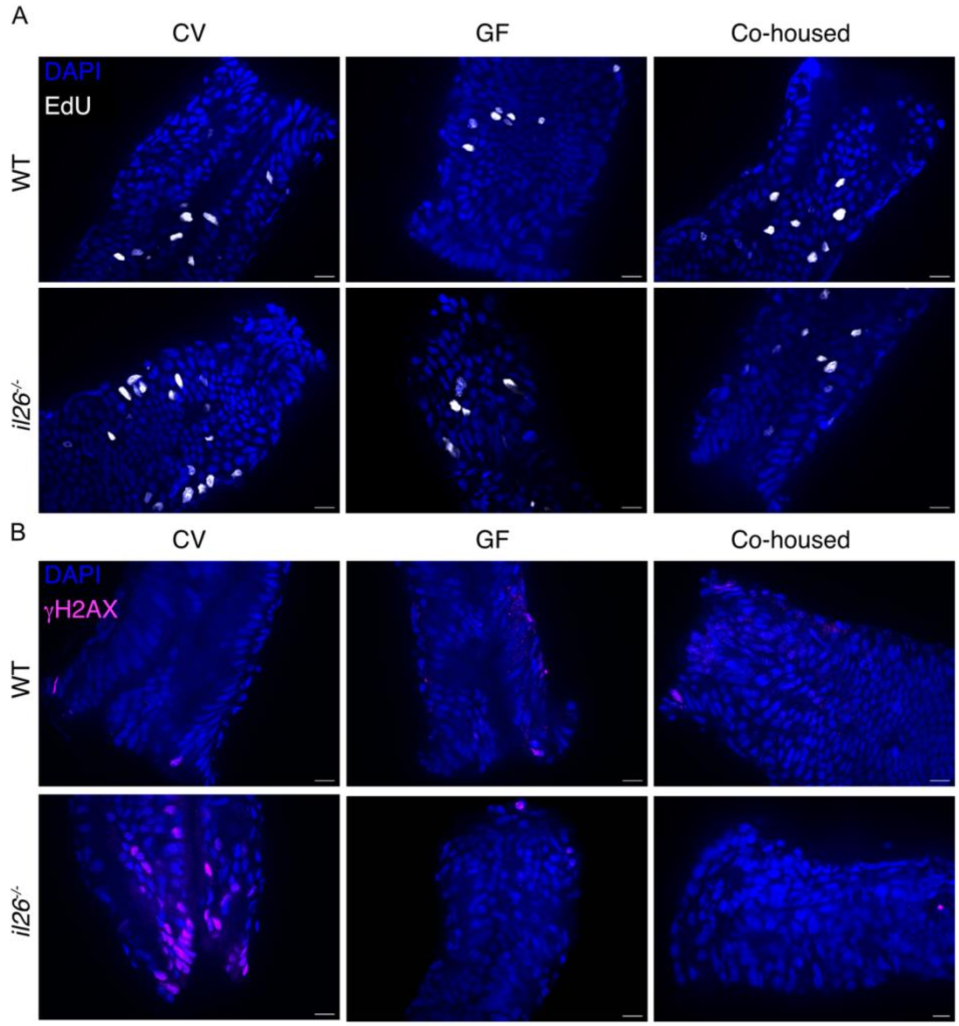

Appendix Figure S6. Immunostaining in WT and *il26*$^{-/-}$ larval guts reared CV, GF, or cohoused. (**A**, **B**) Representative images of EdU (**A**) and γH2AX staining (**B**) in WT and *il26*$^{-/-}$ larval guts reared CV, GF, or cohoused. Scale bars: 10 µm. Source data are available online for this figure.

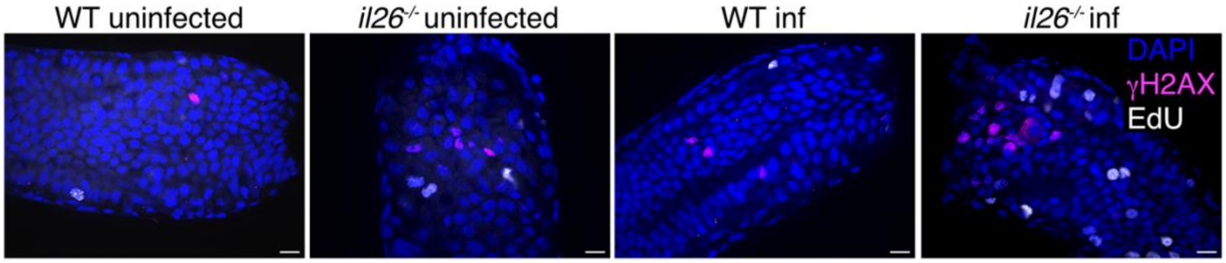

Appendix Figure S7. Immunostaining in WT and *il26$^{-/-}$* larval guts upon *E. tarda* infection. Representative images of EdU and γH2AX staining in WT and *il26$^{-/-}$* at 3 dpi. Scale bars: 10 µm. Source data are available online for this figure.
