# Supplementary material for: IL-26 from innate lymphoid cells regulates early-life gut epithelial homeostasis by shaping microbiota composition
Source: EMBO J. 2025 Oct 22;44(23):6832–56. doi: 10.1038/s44318-025-00588-w (PMC12669248; doi:10.1038/s44318-025-00588-w)
Supplement: Supplementary file 7 — Dataset EV5 [file 44318_2025_588_MOESM7_ESM.zip › Dataset EV5/README.rtf]

This dataset contains relative abundance profiles of bacterial taxa identified by 16S rRNA gene sequencing in wild-type and il26-deficient zebrafish larvae, presented at multiple taxonomic levels from domain to species.
