# Supplementary material for: IL-26 from innate lymphoid cells regulates early-life gut epithelial homeostasis by shaping microbiota composition
Source: EMBO J. 2025 Oct 22;44(23):6832–56. doi: 10.1038/s44318-025-00588-w (PMC12669248; doi:10.1038/s44318-025-00588-w)
Supplement: Supplementary file 8 — Dataset EV6 [file 44318_2025_588_MOESM8_ESM.zip › Dataset EV6/README.rtf]

DEGs in il26KO guts compared to WT  when reared germ-free
