# Supplementary figures and images for: IL-26 from innate lymphoid cells regulates early-life gut epithelial homeostasis by shaping microbiota composition
Source: EMBO J. 2025 Oct 22;44(23):6832–56. doi: 10.1038/s44318-025-00588-w (PMC12669248; doi:10.1038/s44318-025-00588-w)

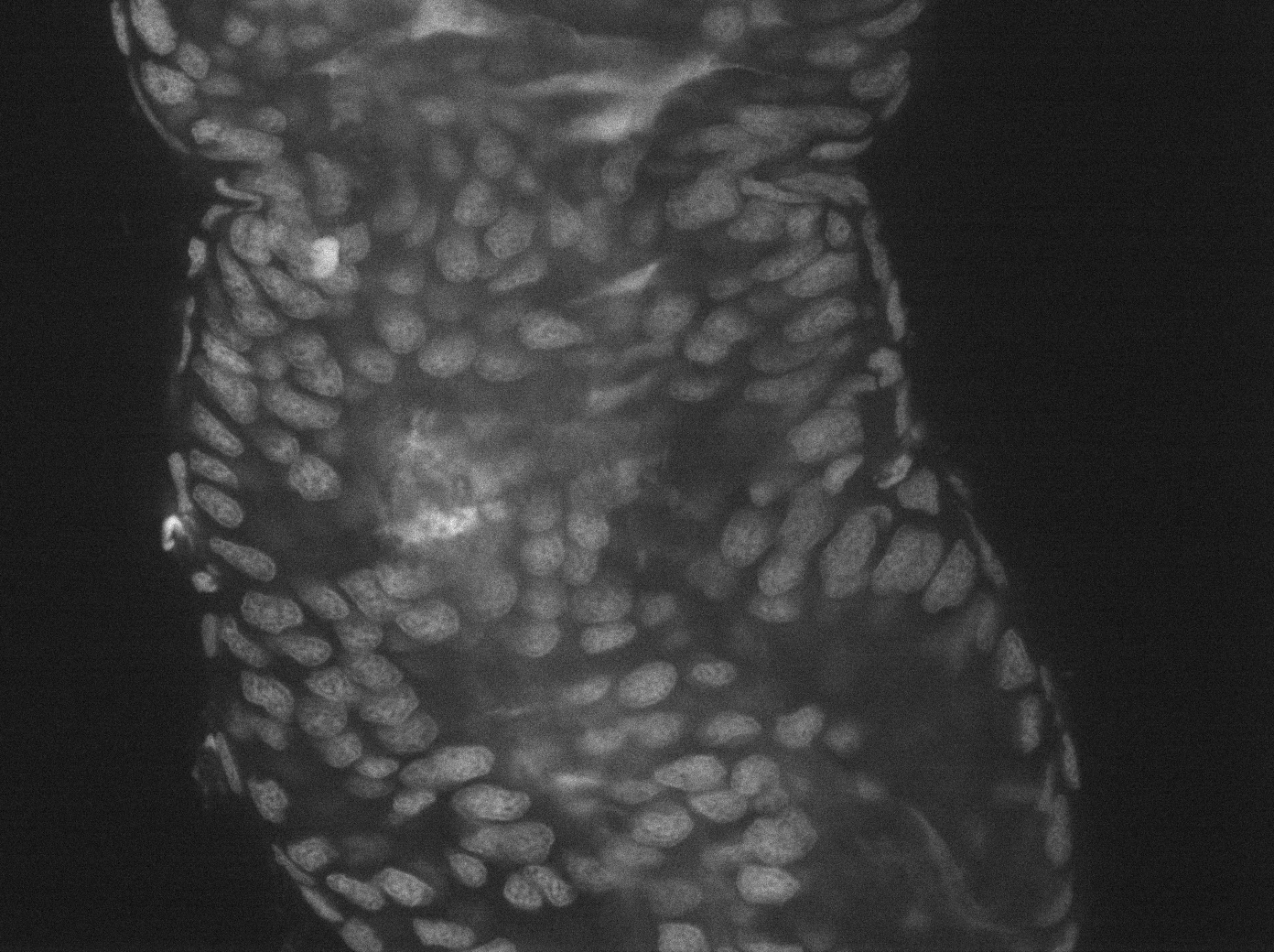

Supplement: Supplementary file 10 — Source data Fig. 1 [file 44318_2025_588_MOESM10_ESM.zip › Figure 1/1E/yH2AX and EdU staining in WT.tif]

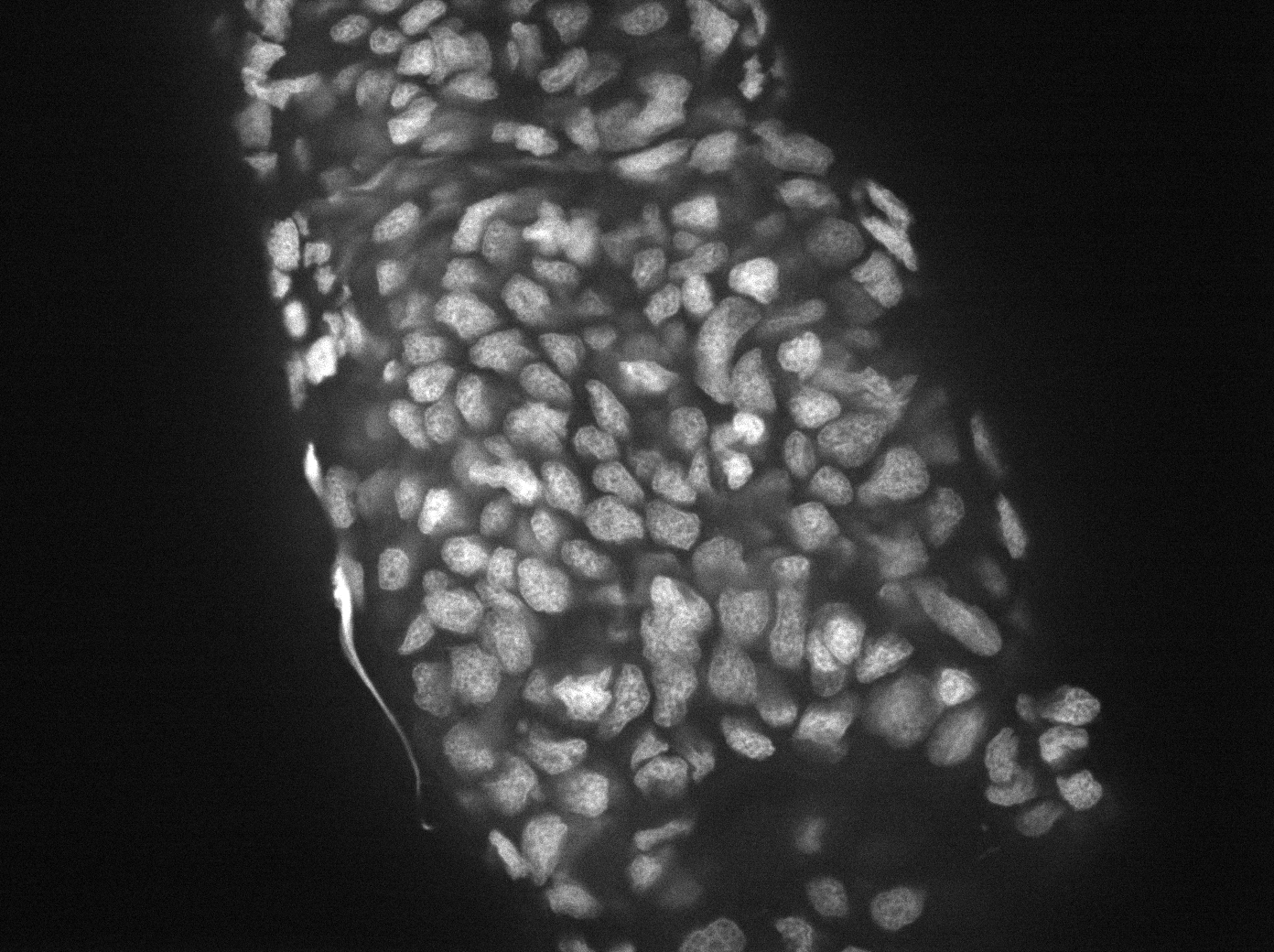

Supplement: Supplementary file 10 — Source data Fig. 1 [file 44318_2025_588_MOESM10_ESM.zip › Figure 1/1E/yH2AX and EdU staining in il26ko.tif]

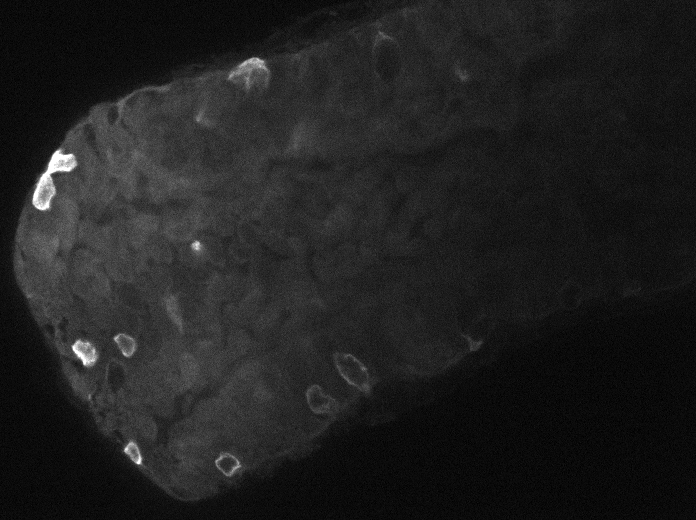

Supplement: Supplementary file 11 — Source data Fig. 3 [file 44318_2025_588_MOESM11_ESM.zip › Figure 3/3C/yH2AX and 2F11 staining in il26ko.tif]

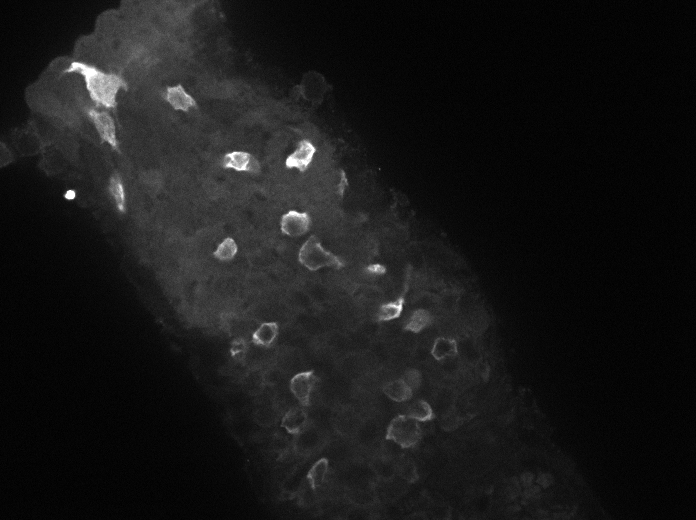

Supplement: Supplementary file 11 — Source data Fig. 3 [file 44318_2025_588_MOESM11_ESM.zip › Figure 3/3C/yH2AX and 2F11 staining in WT.tif]

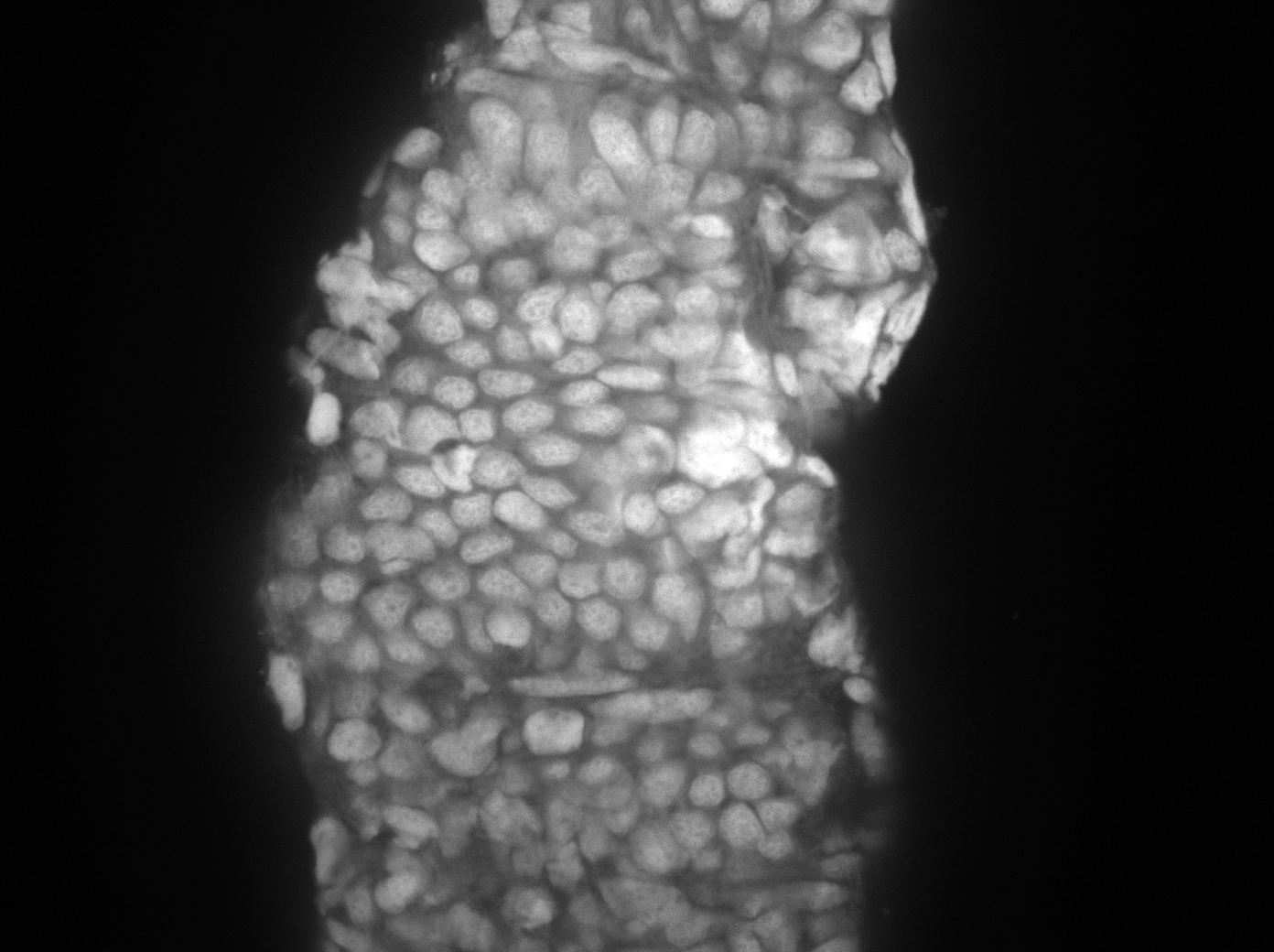

Supplement: Supplementary file 11 — Source data Fig. 3 [file 44318_2025_588_MOESM11_ESM.zip › Figure 3/3A/yH2AX staining in cldn15-GFP.tif]

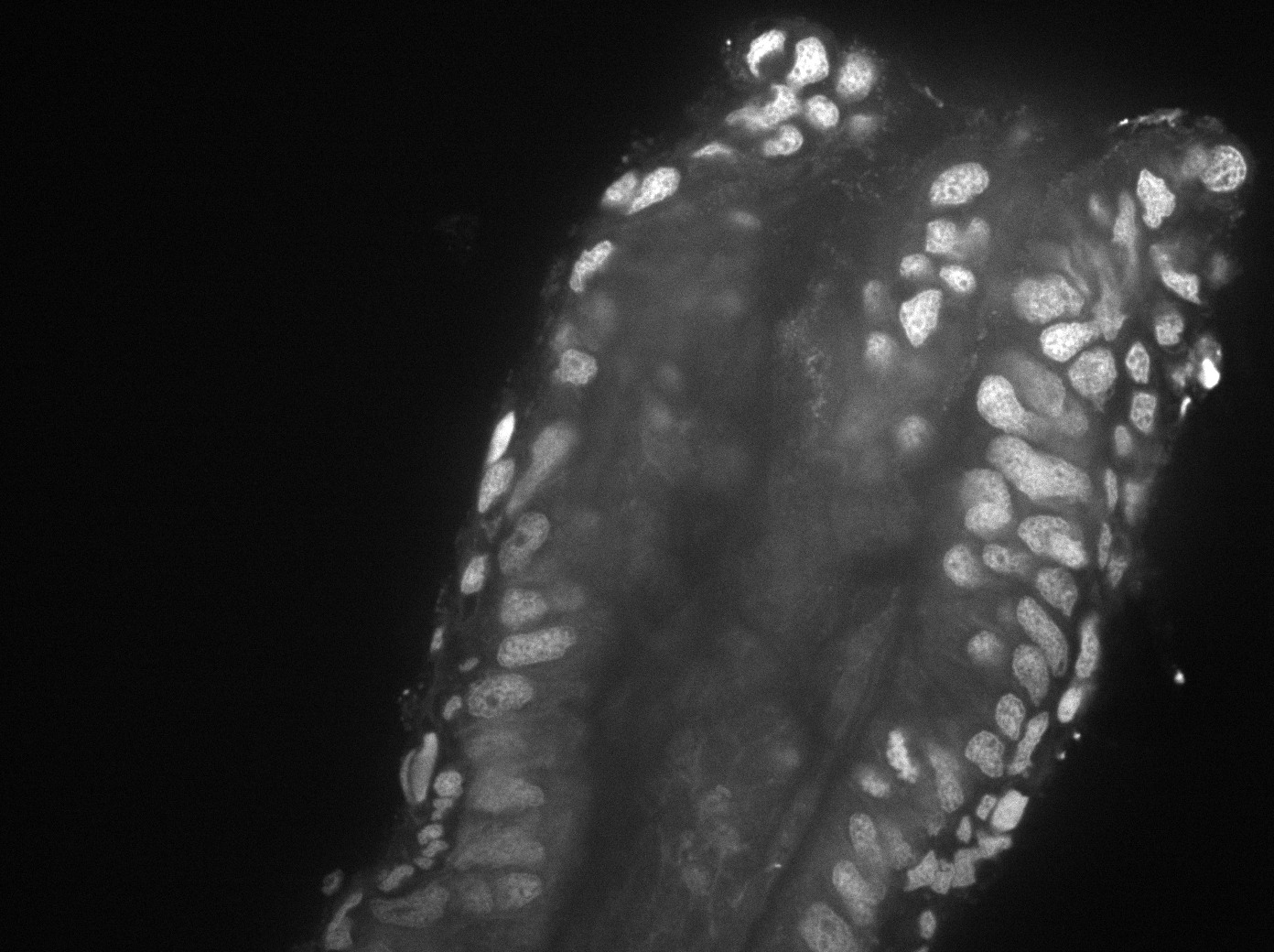

Supplement: Supplementary file 11 — Source data Fig. 3 [file 44318_2025_588_MOESM11_ESM.zip › Figure 3/3A/yH2AX staining in cldn15-GFP il26ko.tif]

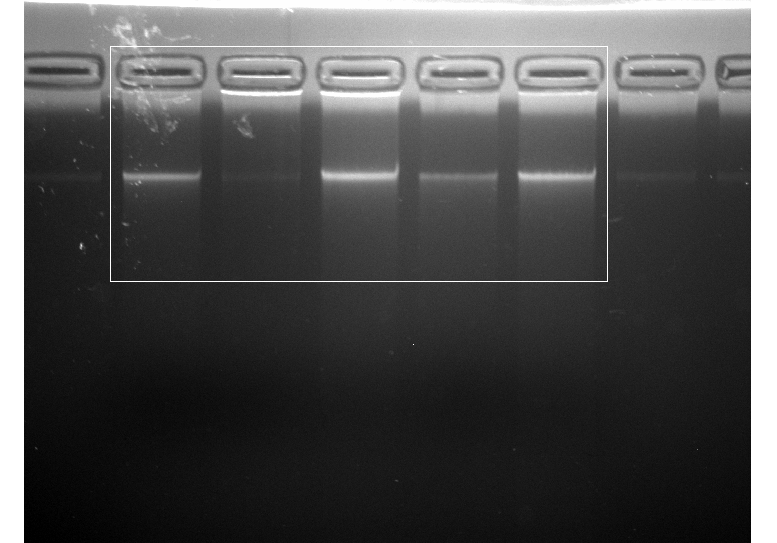

Supplement: Supplementary file 13 — Source data Fig. 5 [file 44318_2025_588_MOESM13_ESM.zip › Figure 5/5A/Agarose gel of IL26-DNA complexes.tif]

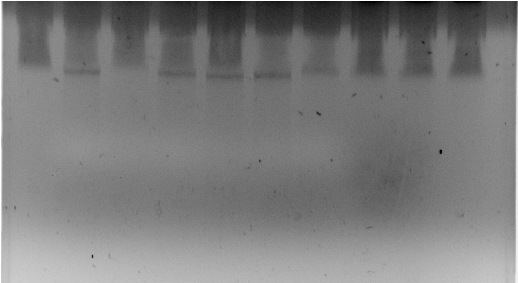

Supplement: Supplementary file 13 — Source data Fig. 5 [file 44318_2025_588_MOESM13_ESM.zip › Figure 5/5B/Agarose gel of IL26-DNA complexes.tif]

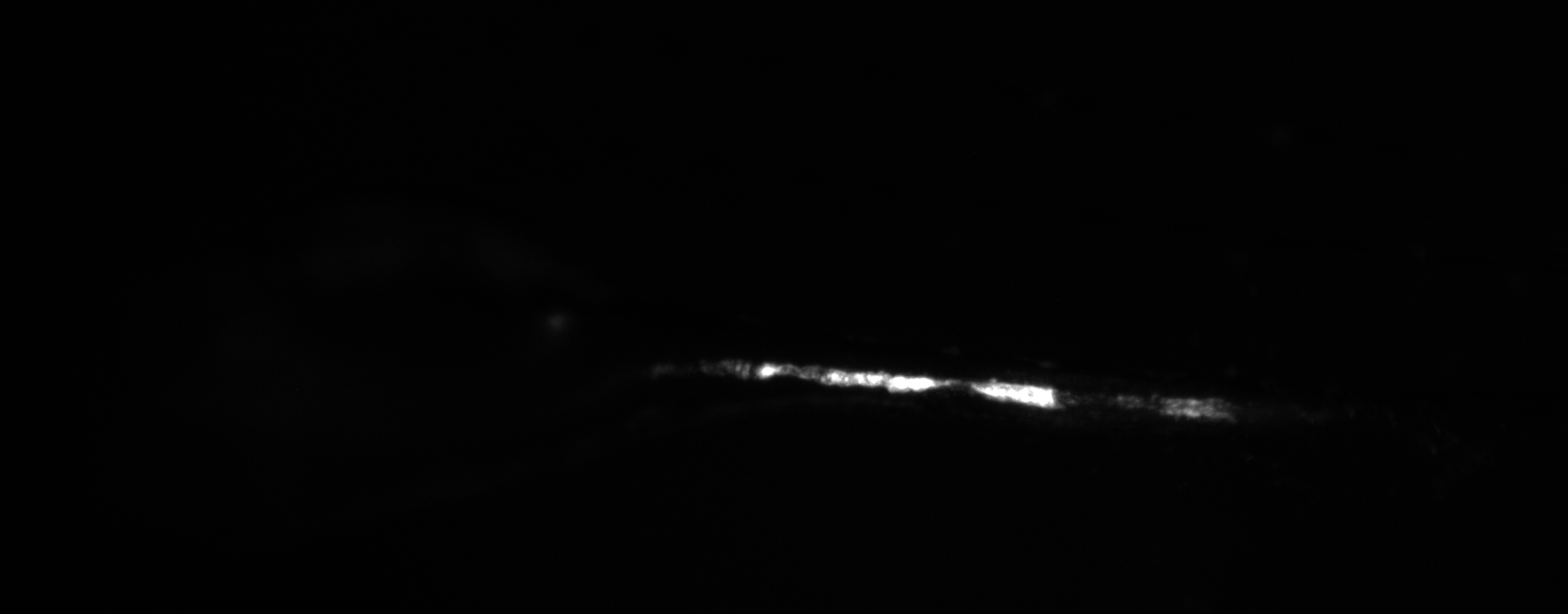

Supplement: Supplementary file 15 — Source data Fig. 7 [file 44318_2025_588_MOESM15_ESM.zip › Figure 7/7B/il26ko infected with Etarda-mCherry.tif]

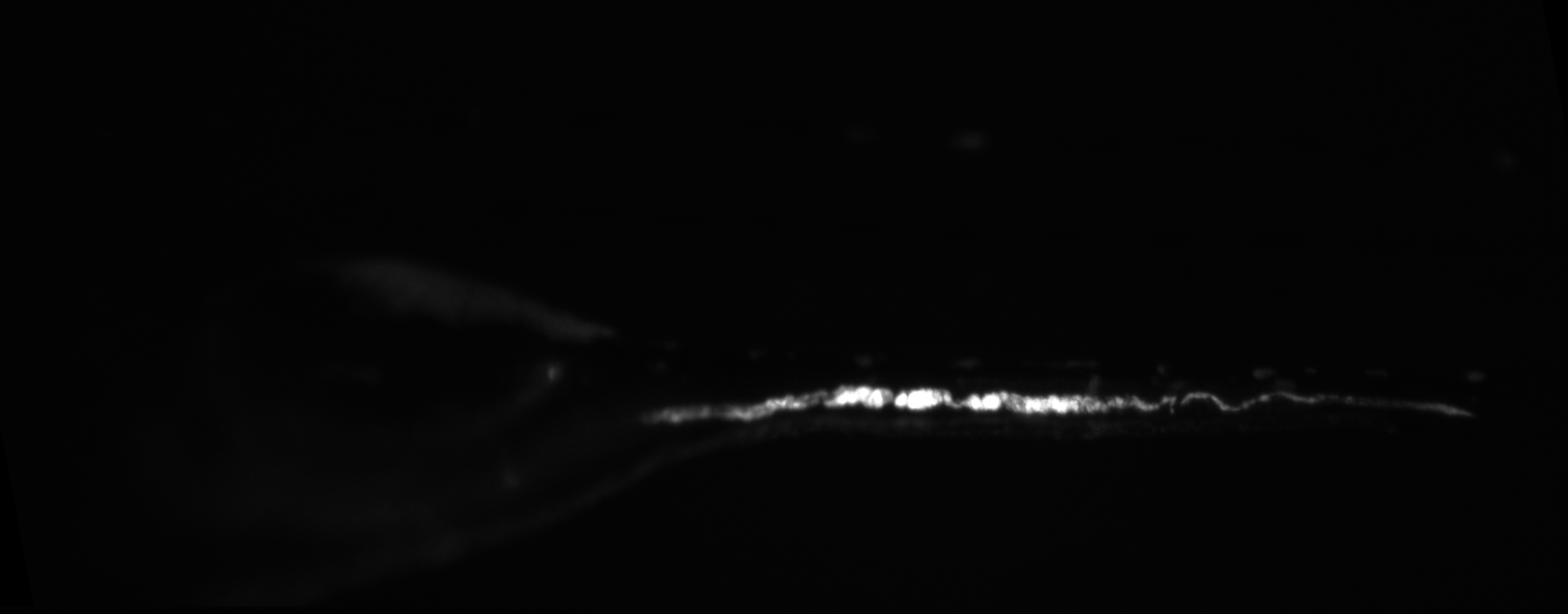

Supplement: Supplementary file 15 — Source data Fig. 7 [file 44318_2025_588_MOESM15_ESM.zip › Figure 7/7B/WT infected with Etarda-mCherry.tif]

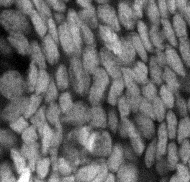

Supplement: Supplementary file 16 — Source data Fig. 8 [file 44318_2025_588_MOESM16_ESM.zip › Figure 8/8F/il26 and nitr9 HCR.tif]

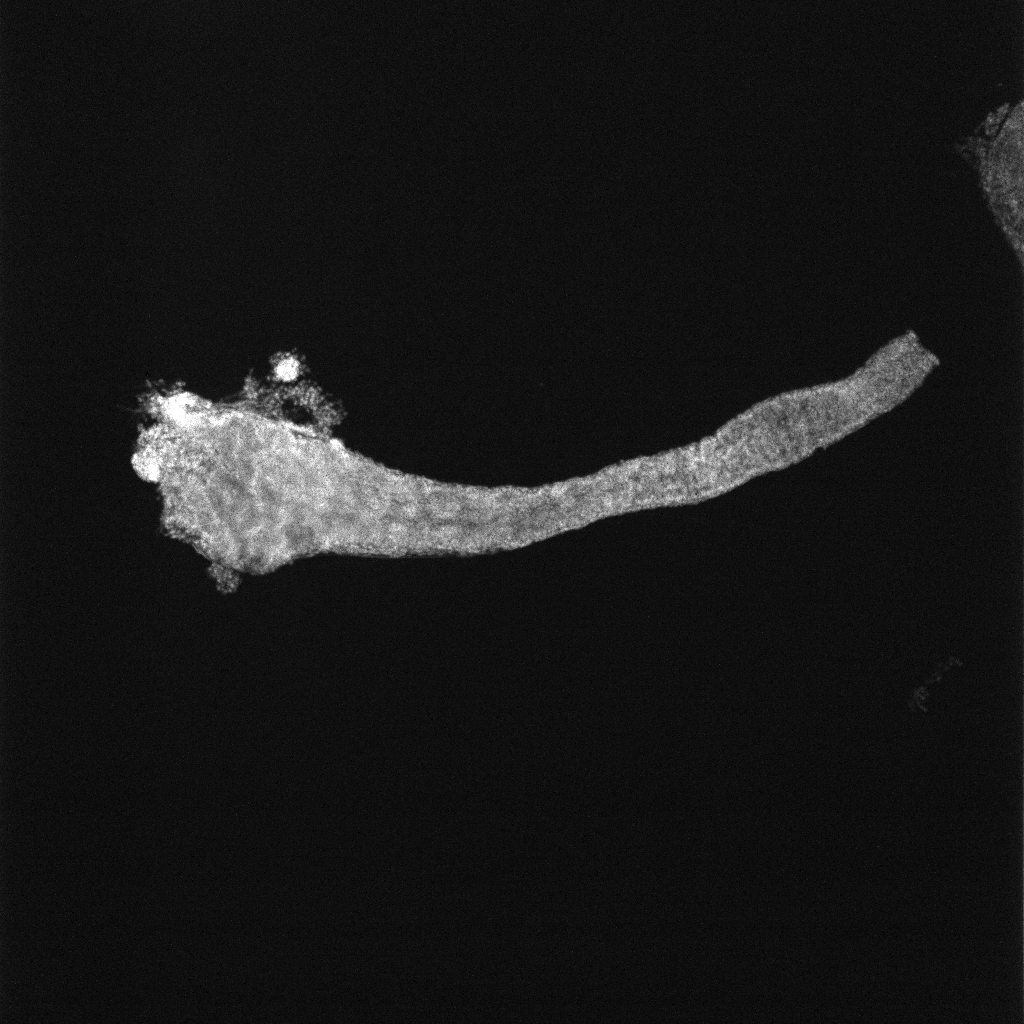

Supplement: Supplementary file 17 — Figure EV2 Source Data [file 44318_2025_588_MOESM17_ESM.zip › Figure EV2/EV2C/yH2AX staining in WT full guts.tif]

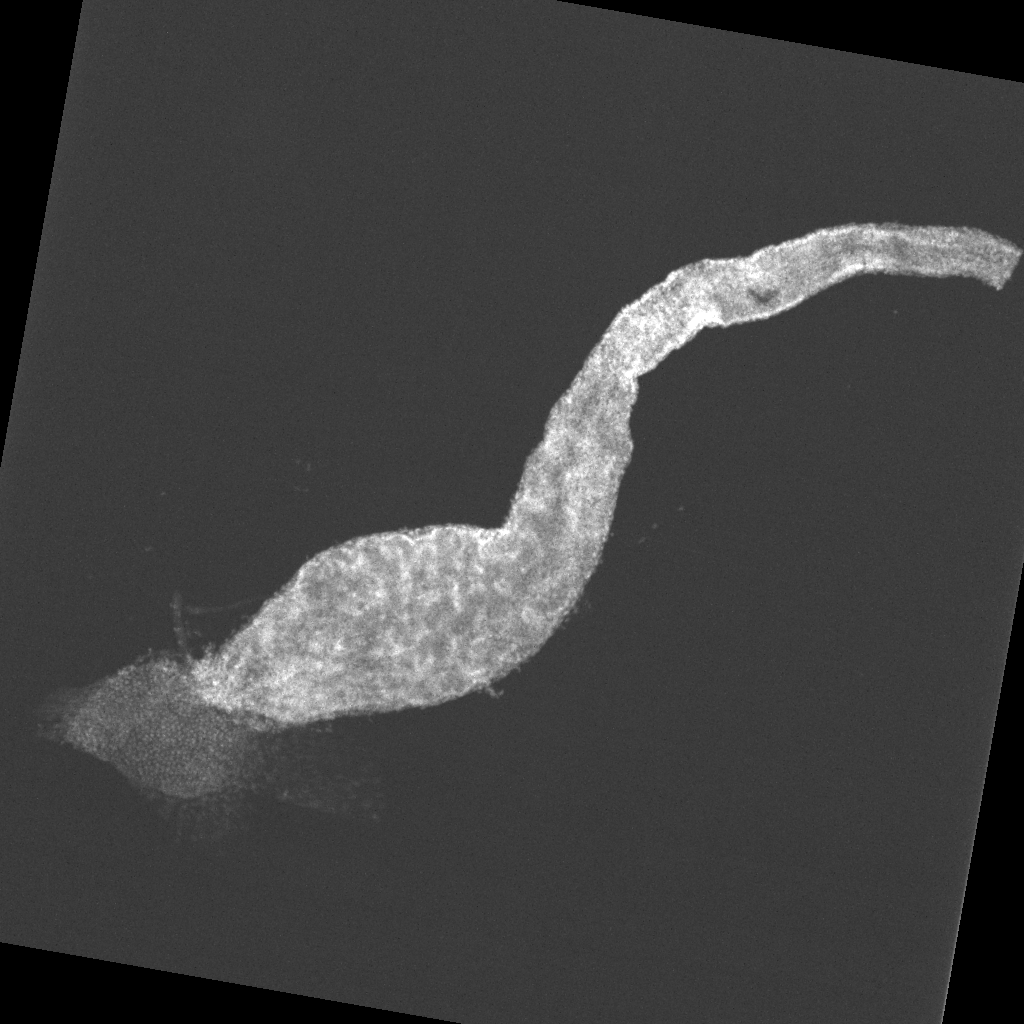

Supplement: Supplementary file 17 — Figure EV2 Source Data [file 44318_2025_588_MOESM17_ESM.zip › Figure EV2/EV2C/yH2AX staining in il26ko full guts.tif]

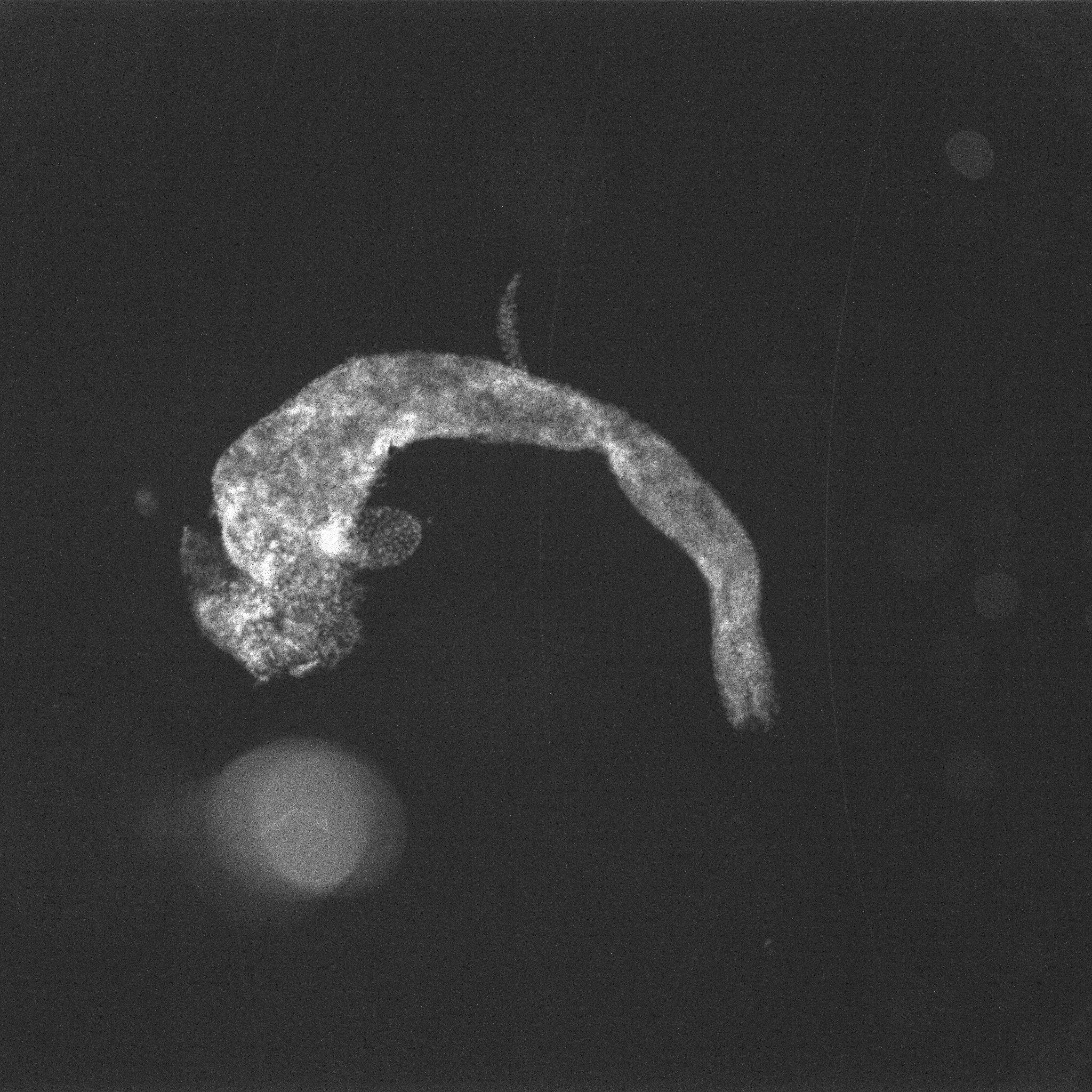

Supplement: Supplementary file 17 — Figure EV2 Source Data [file 44318_2025_588_MOESM17_ESM.zip › Figure EV2/EV2A/EdU staining in il26ko full guts.tif.tif]

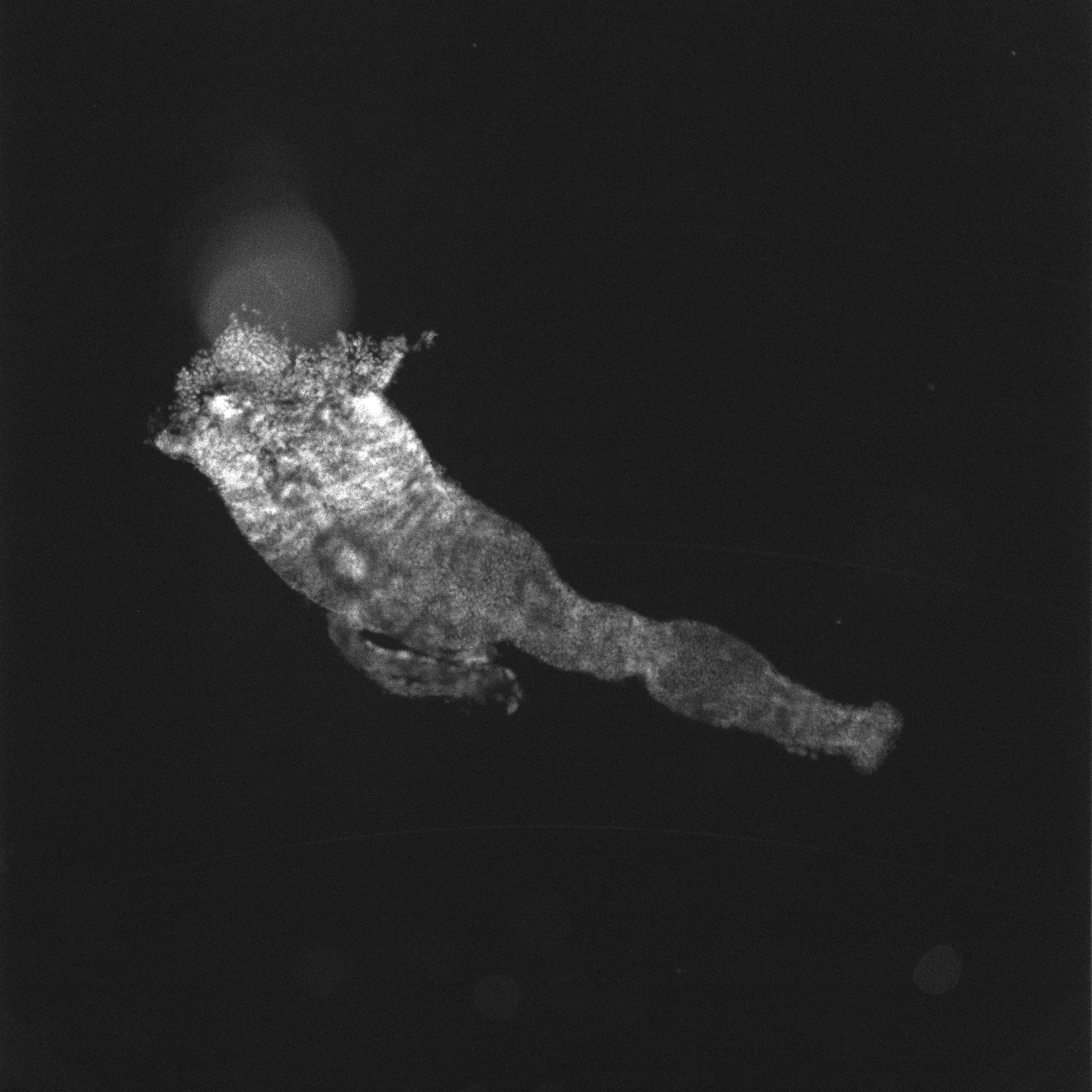

Supplement: Supplementary file 17 — Figure EV2 Source Data [file 44318_2025_588_MOESM17_ESM.zip › Figure EV2/EV2A/EdU staining in WT full guts.tif]

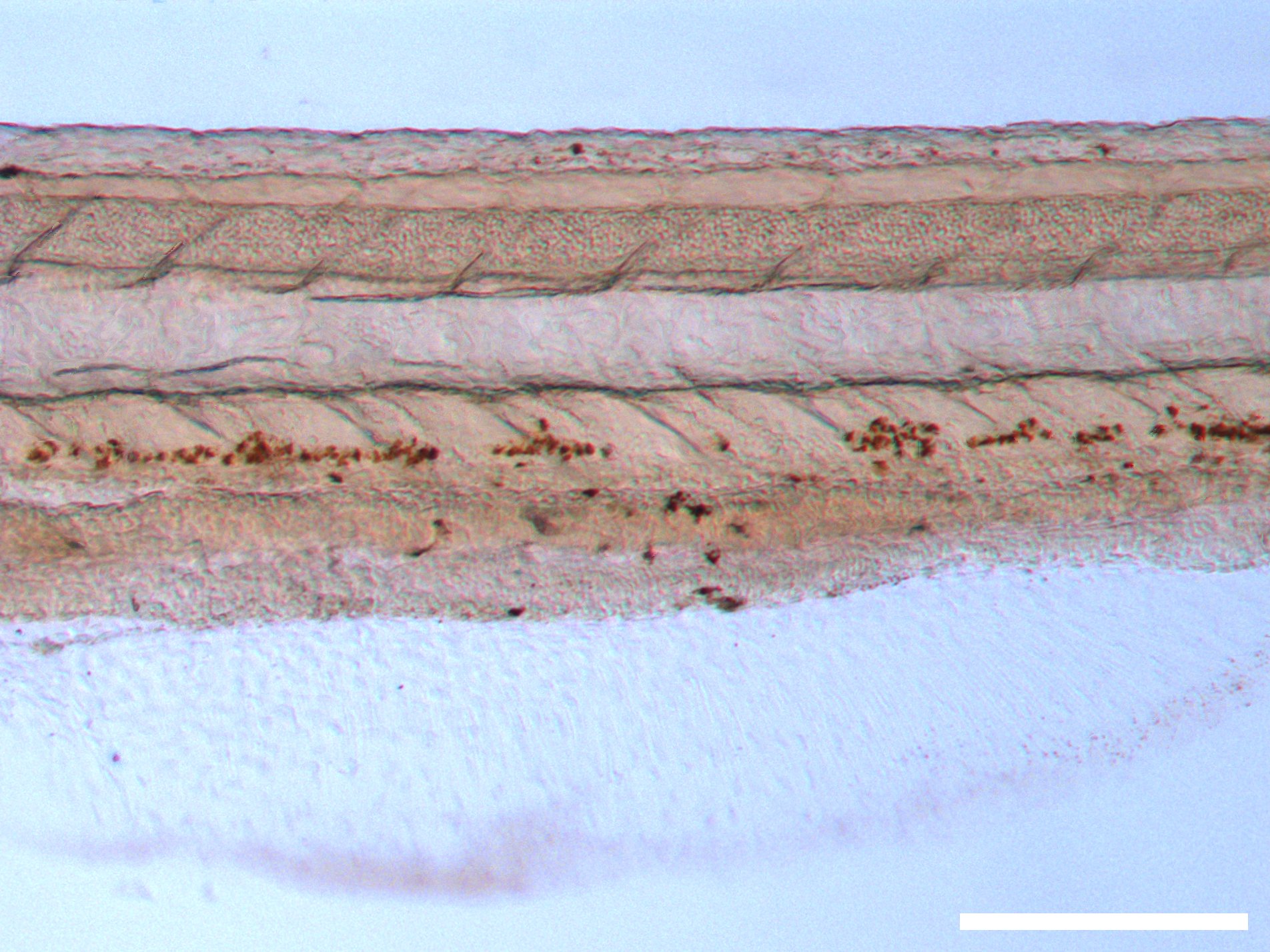

Supplement: Supplementary file 18 — Figure EV3 Source Data [file 44318_2025_588_MOESM18_ESM.zip › Figure EV3/EV3F/Neutrophil staining in il26ko.jpg]

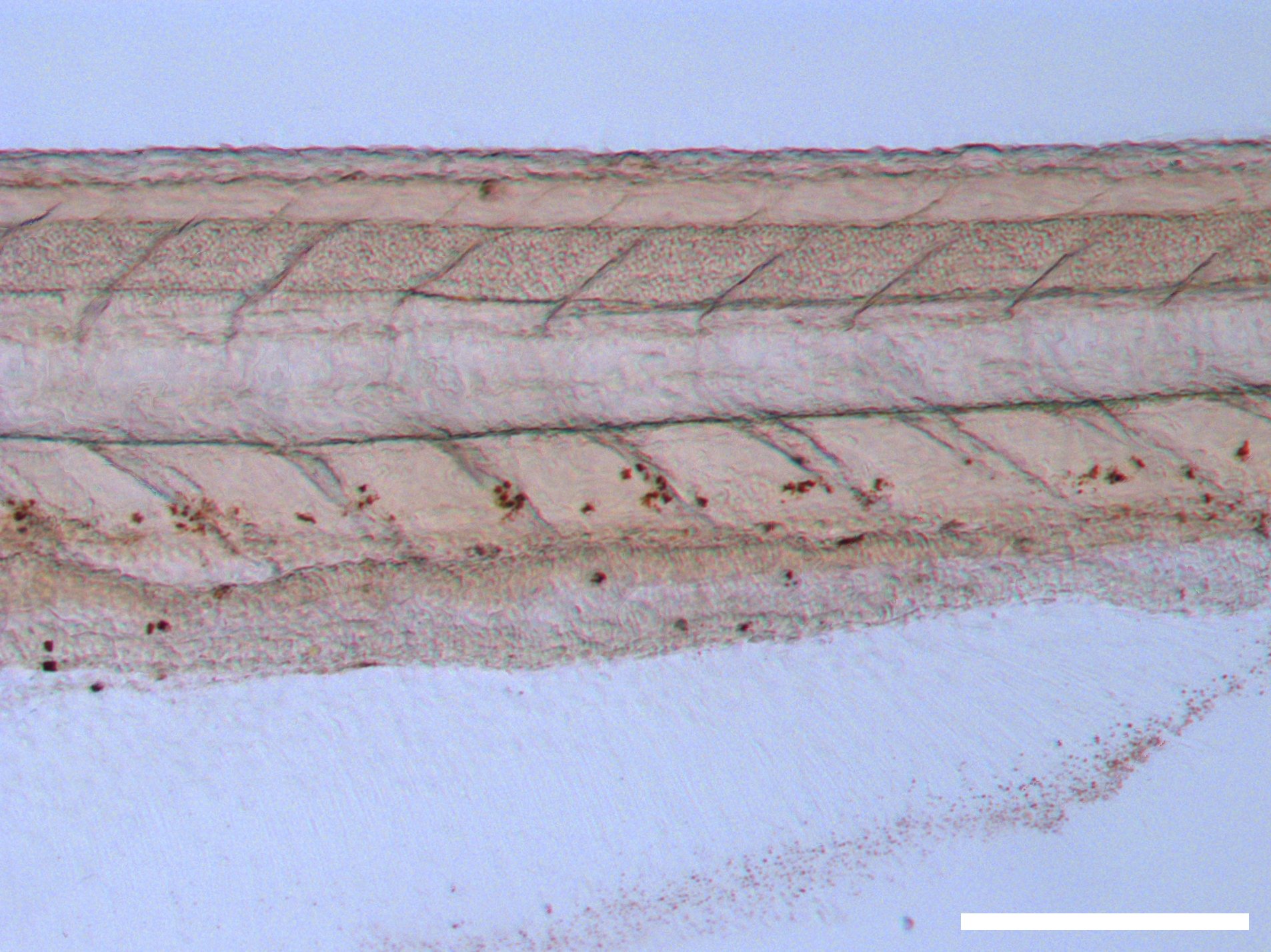

Supplement: Supplementary file 18 — Figure EV3 Source Data [file 44318_2025_588_MOESM18_ESM.zip › Figure EV3/EV3F/Neutrophil staining in WT.jpg]

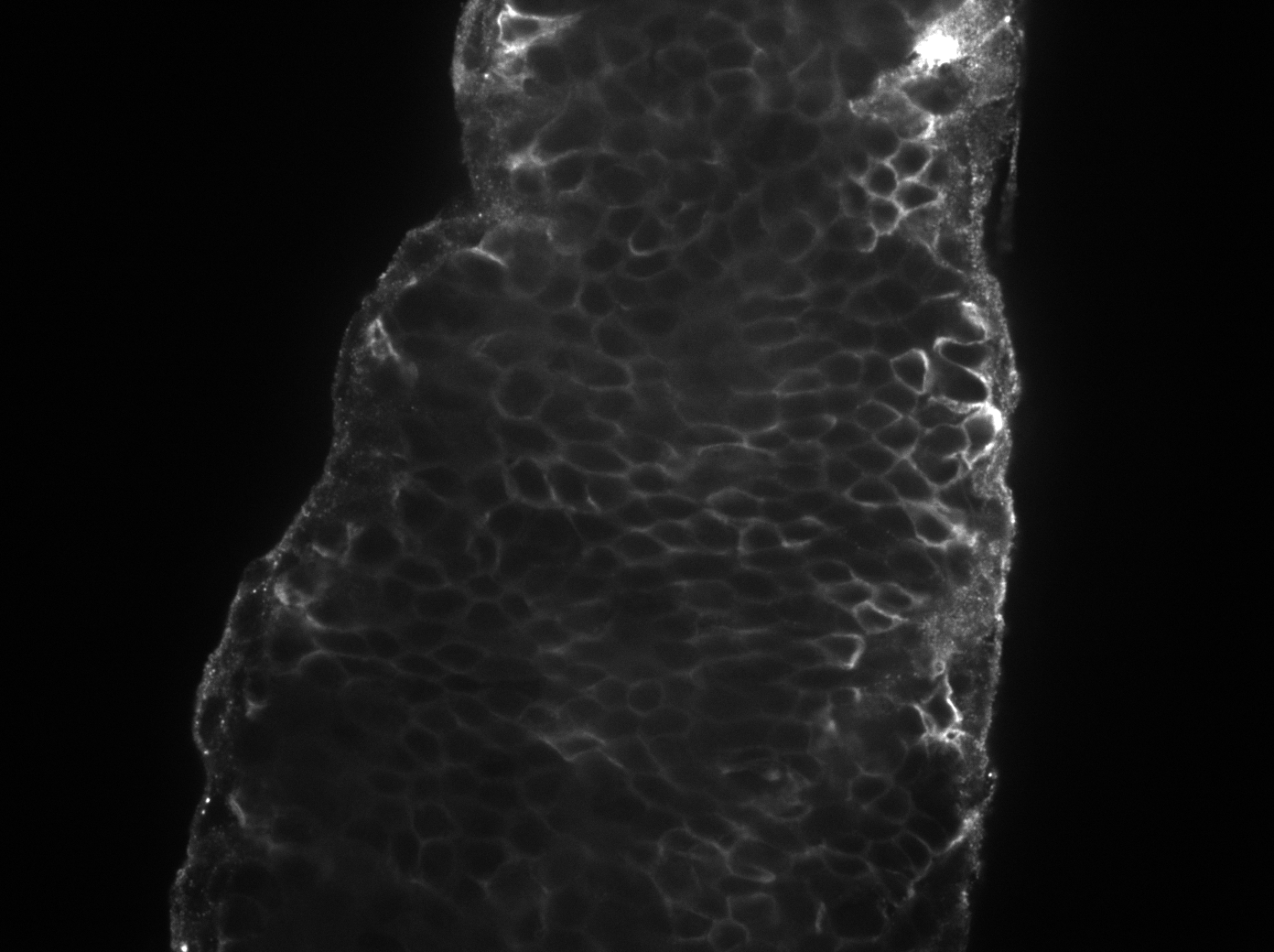

Supplement: Supplementary file 18 — Figure EV3 Source Data [file 44318_2025_588_MOESM18_ESM.zip › Figure EV3/EV3A/EdU staining in cldn15a-GFP in il26ko.tif]

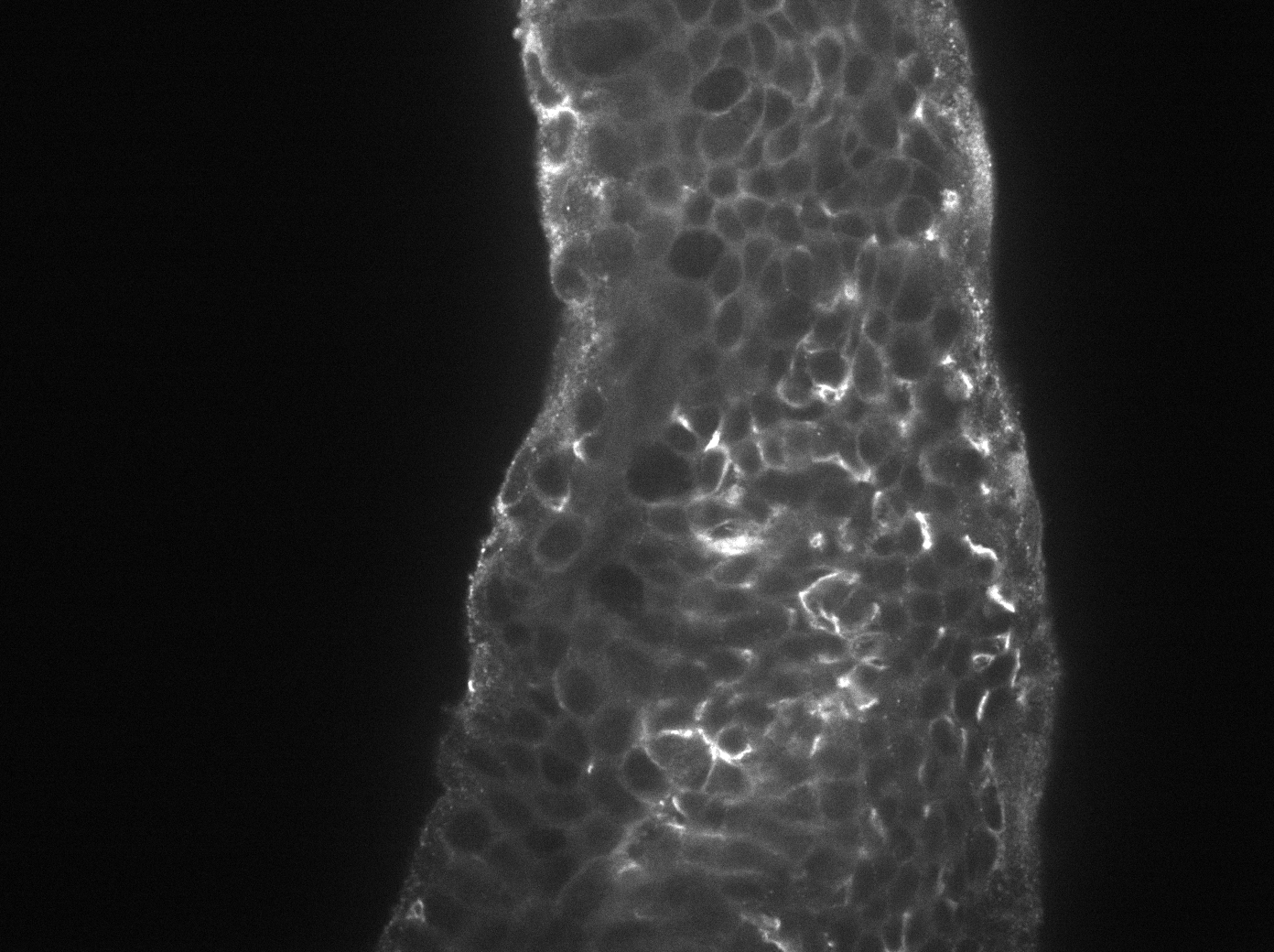

Supplement: Supplementary file 18 — Figure EV3 Source Data [file 44318_2025_588_MOESM18_ESM.zip › Figure EV3/EV3A/EdU staining in cldn15a-GFP.tif]

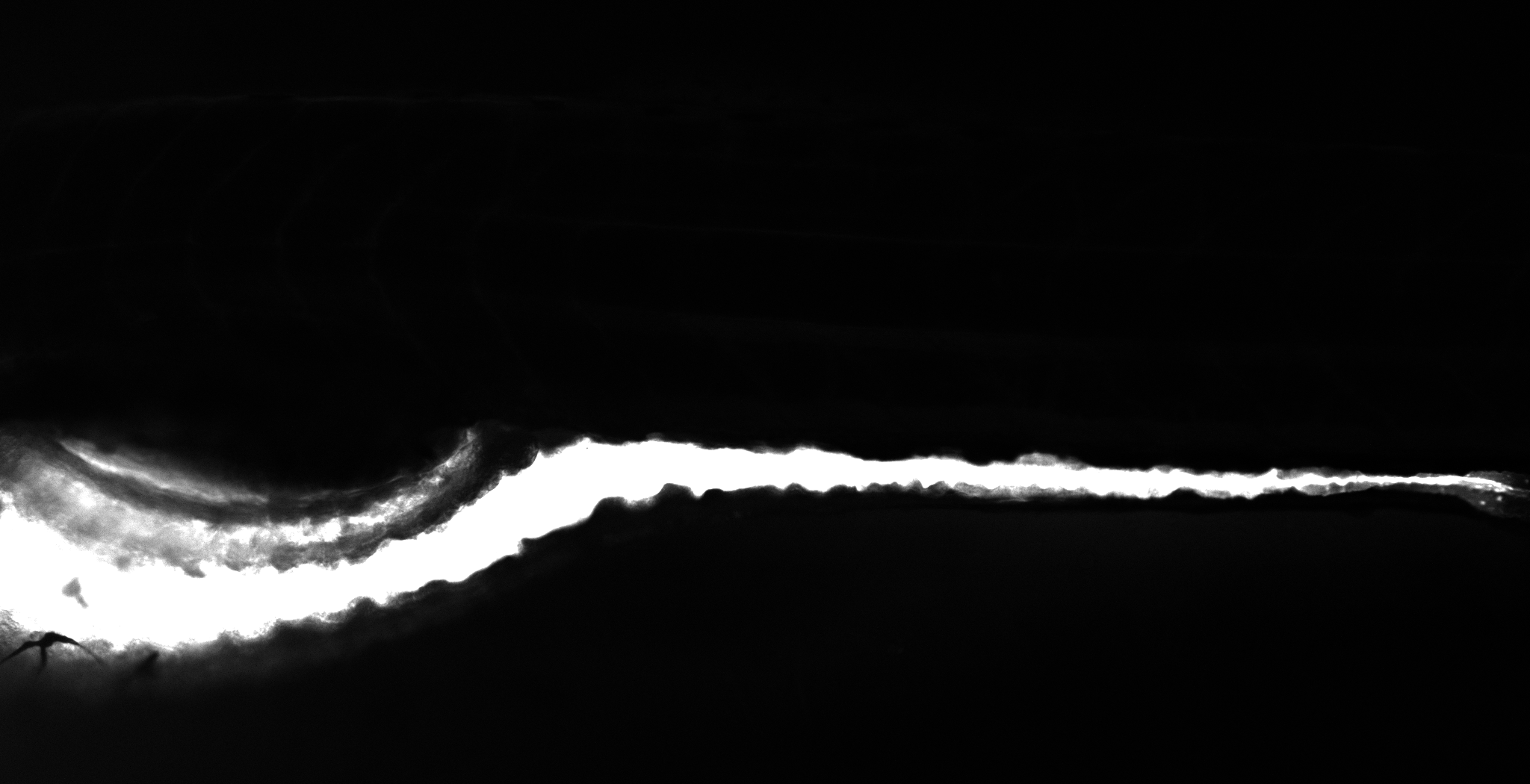

Supplement: Supplementary file 18 — Figure EV3 Source Data [file 44318_2025_588_MOESM18_ESM.zip › Figure EV3/EV3D/dextran in WT.tif]

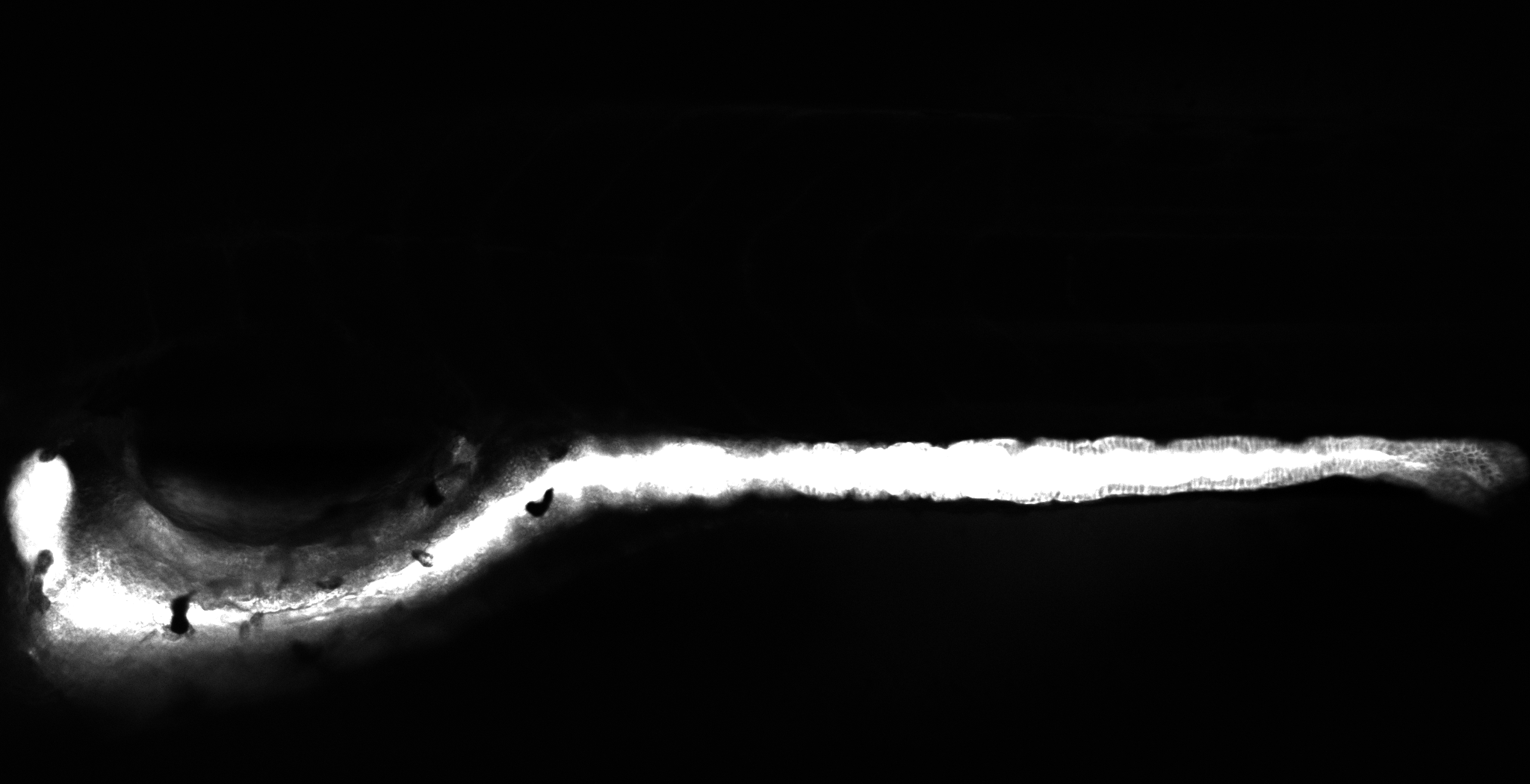

Supplement: Supplementary file 18 — Figure EV3 Source Data [file 44318_2025_588_MOESM18_ESM.zip › Figure EV3/EV3D/Dextran in il26ko.tif]

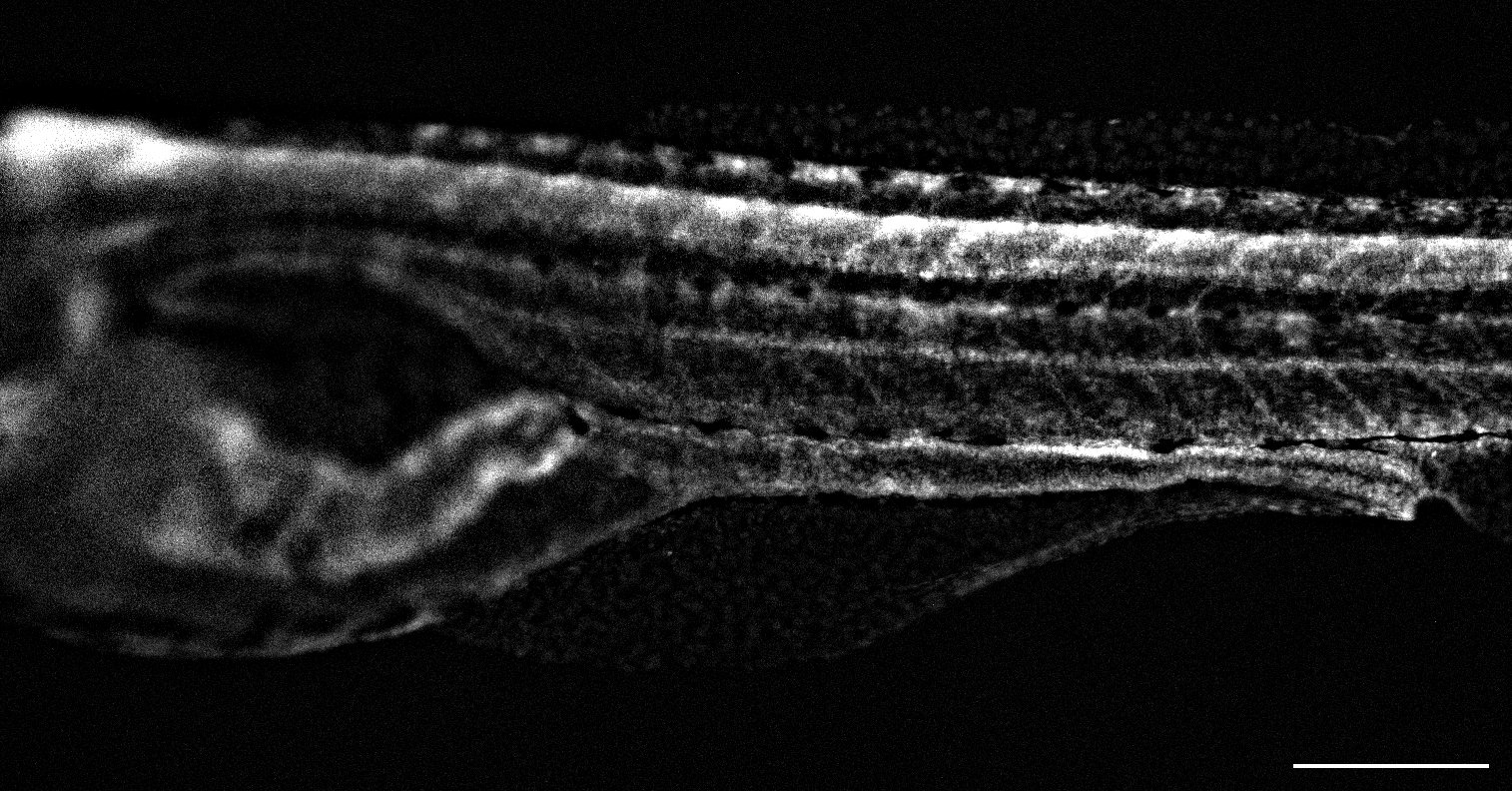

Supplement: Supplementary file 18 — Figure EV3 Source Data [file 44318_2025_588_MOESM18_ESM.zip › Figure EV3/EV3B/DAPI in WT to quantifu gut length.jpg]

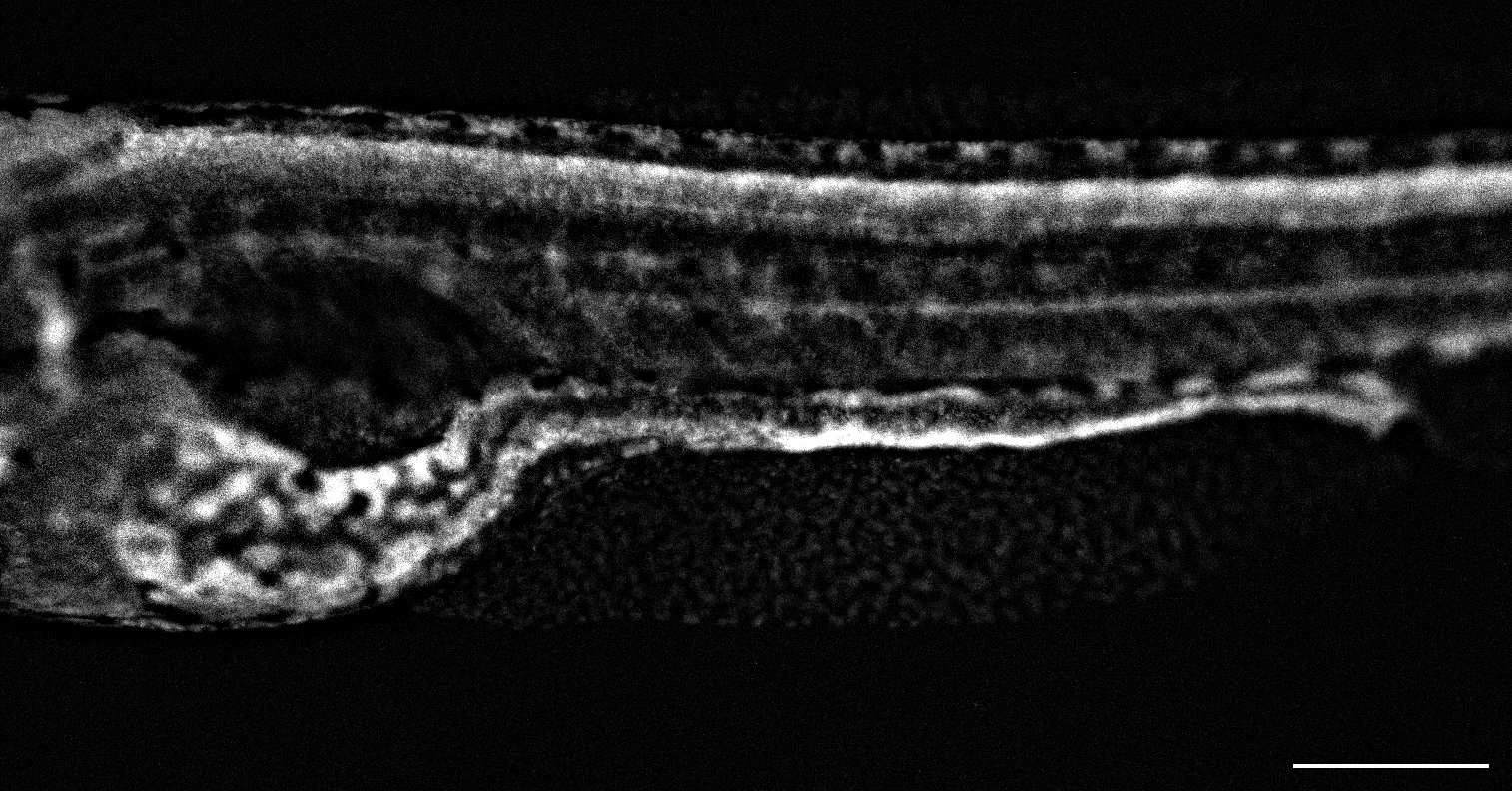

Supplement: Supplementary file 18 — Figure EV3 Source Data [file 44318_2025_588_MOESM18_ESM.zip › Figure EV3/EV3B/DAPI in il26ko to quantifu gut length.jpg]

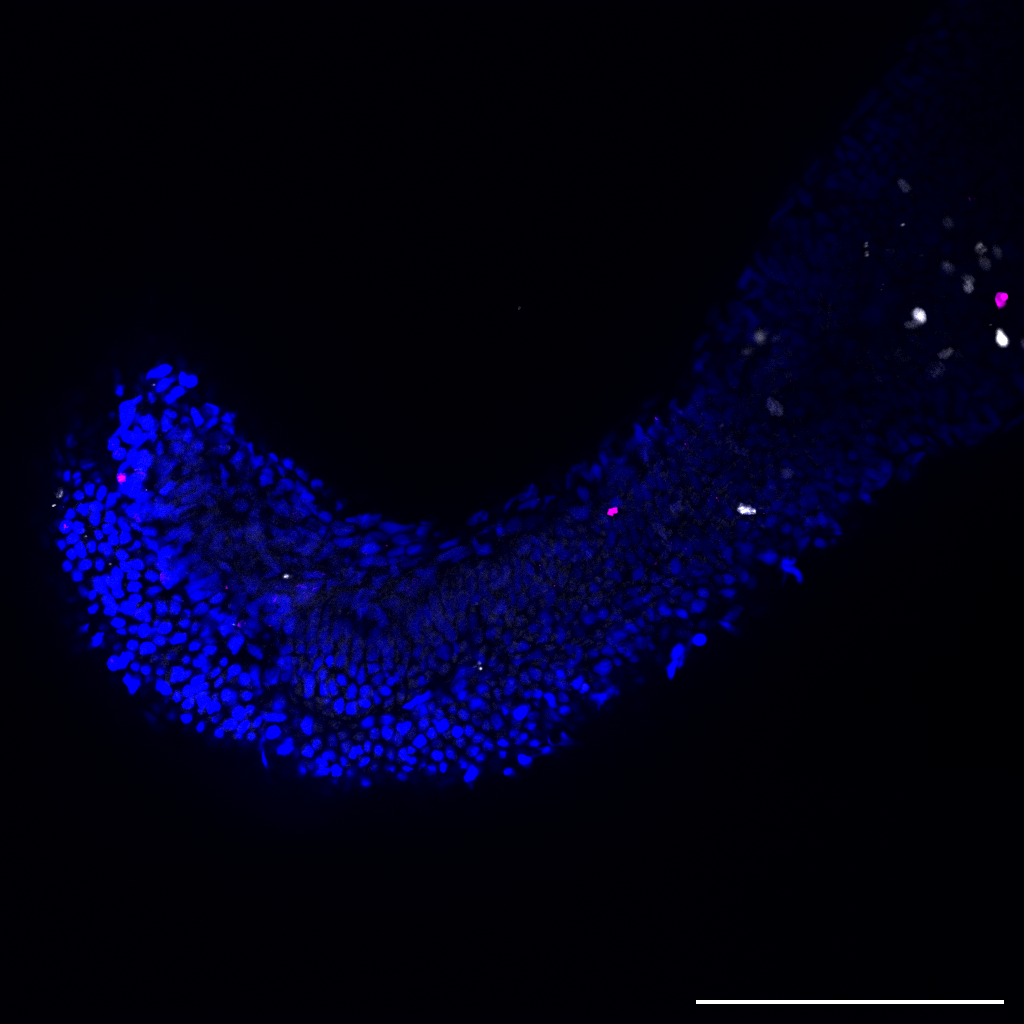

Supplement: Supplementary file 19 — Figure EV4 Source Data [file 44318_2025_588_MOESM19_ESM.zip › Figure EV4/EV4A/yH2AX and EdU staining in il26ko juvenile guts.tif (RGB)-1.tif]

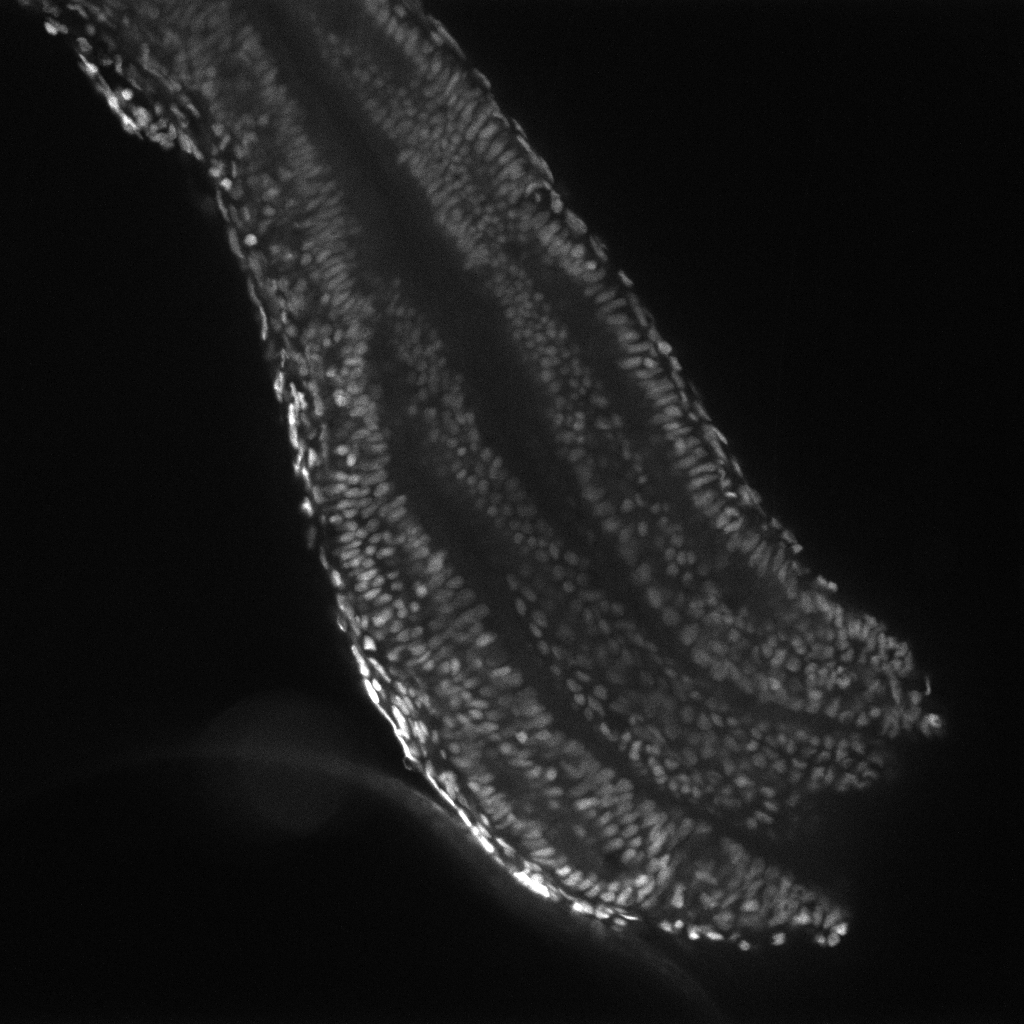

Supplement: Supplementary file 19 — Figure EV4 Source Data [file 44318_2025_588_MOESM19_ESM.zip › Figure EV4/EV4A/yH2AX and EdU staining in WT juvenile guts.tif]

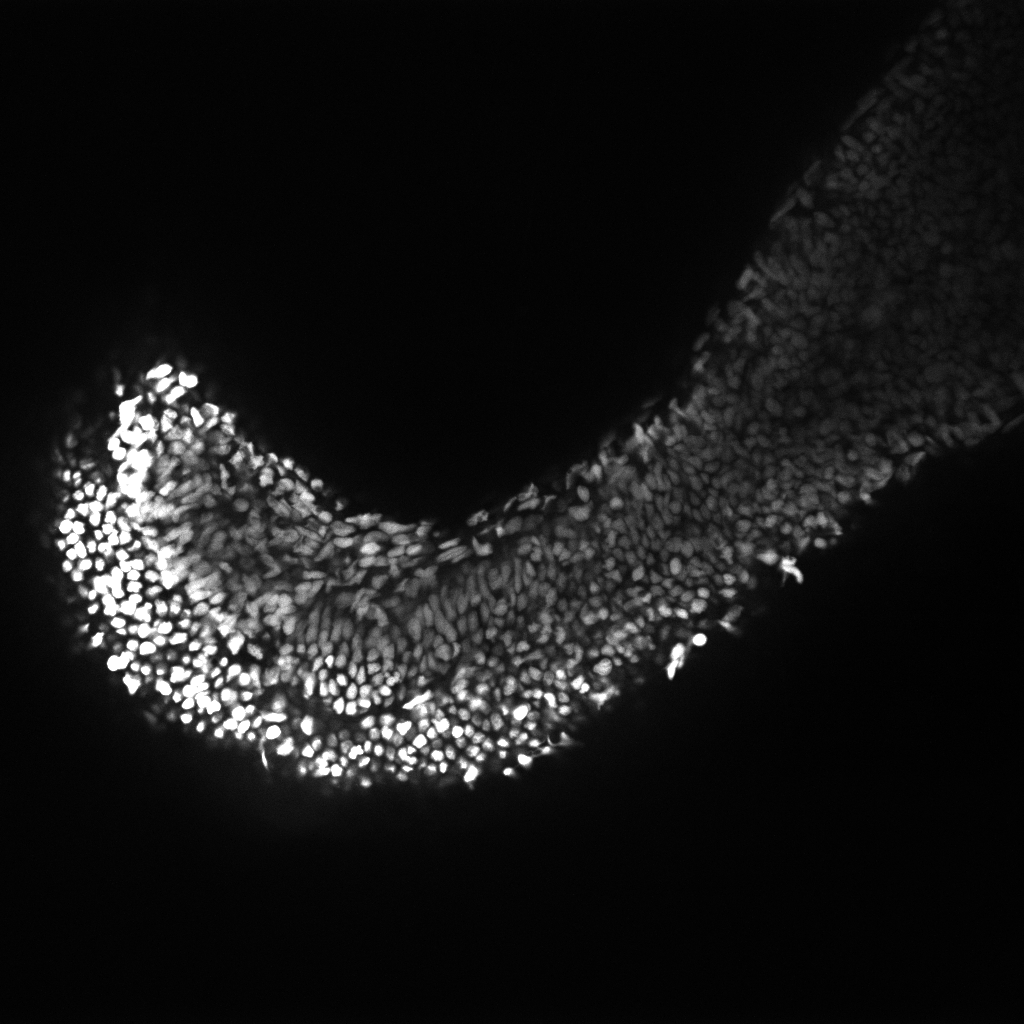

Supplement: Supplementary file 19 — Figure EV4 Source Data [file 44318_2025_588_MOESM19_ESM.zip › Figure EV4/EV4A/yH2AX and EdU staining in il26ko juvenile guts.tif]

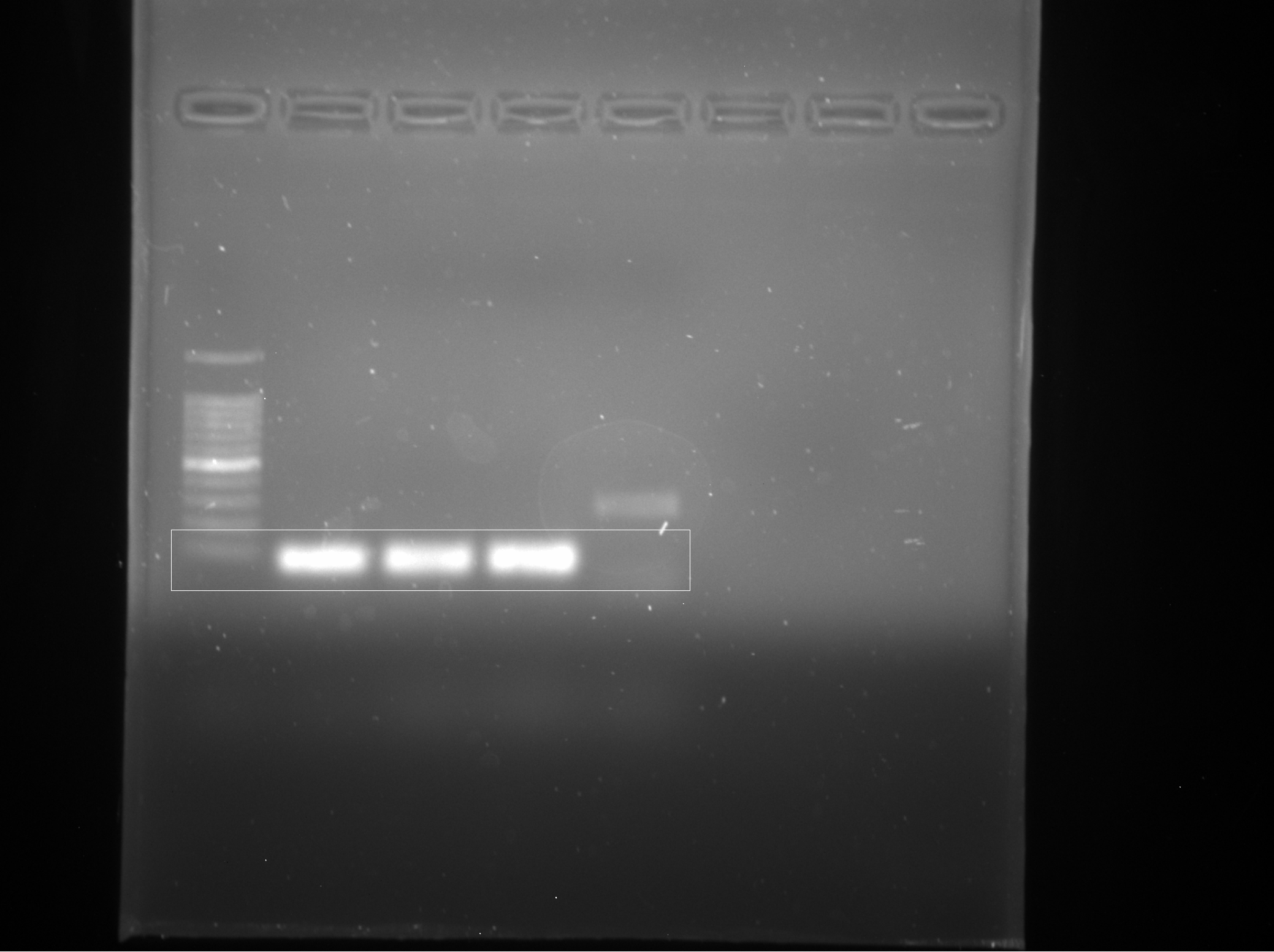

Supplement: Supplementary file 21 — Appendix Figure Source Data [file 44318_2025_588_MOESM21_ESM.zip › Figure S1/S1A/Agarose gel of il26 transcript.tif]

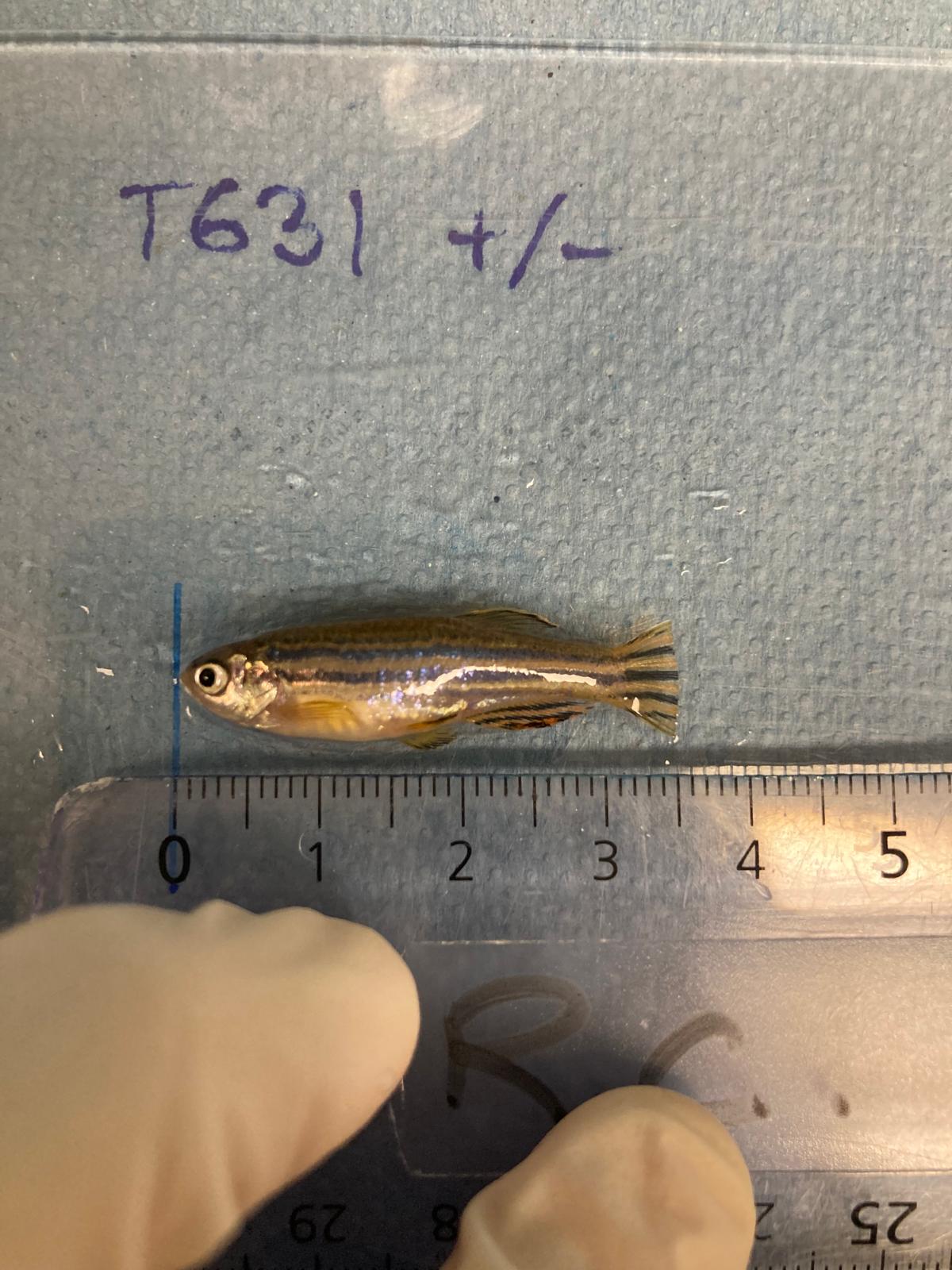

Supplement: Supplementary file 21 — Appendix Figure Source Data [file 44318_2025_588_MOESM21_ESM.zip › Figure S4/S4A/Adult il26+:-.jpeg]

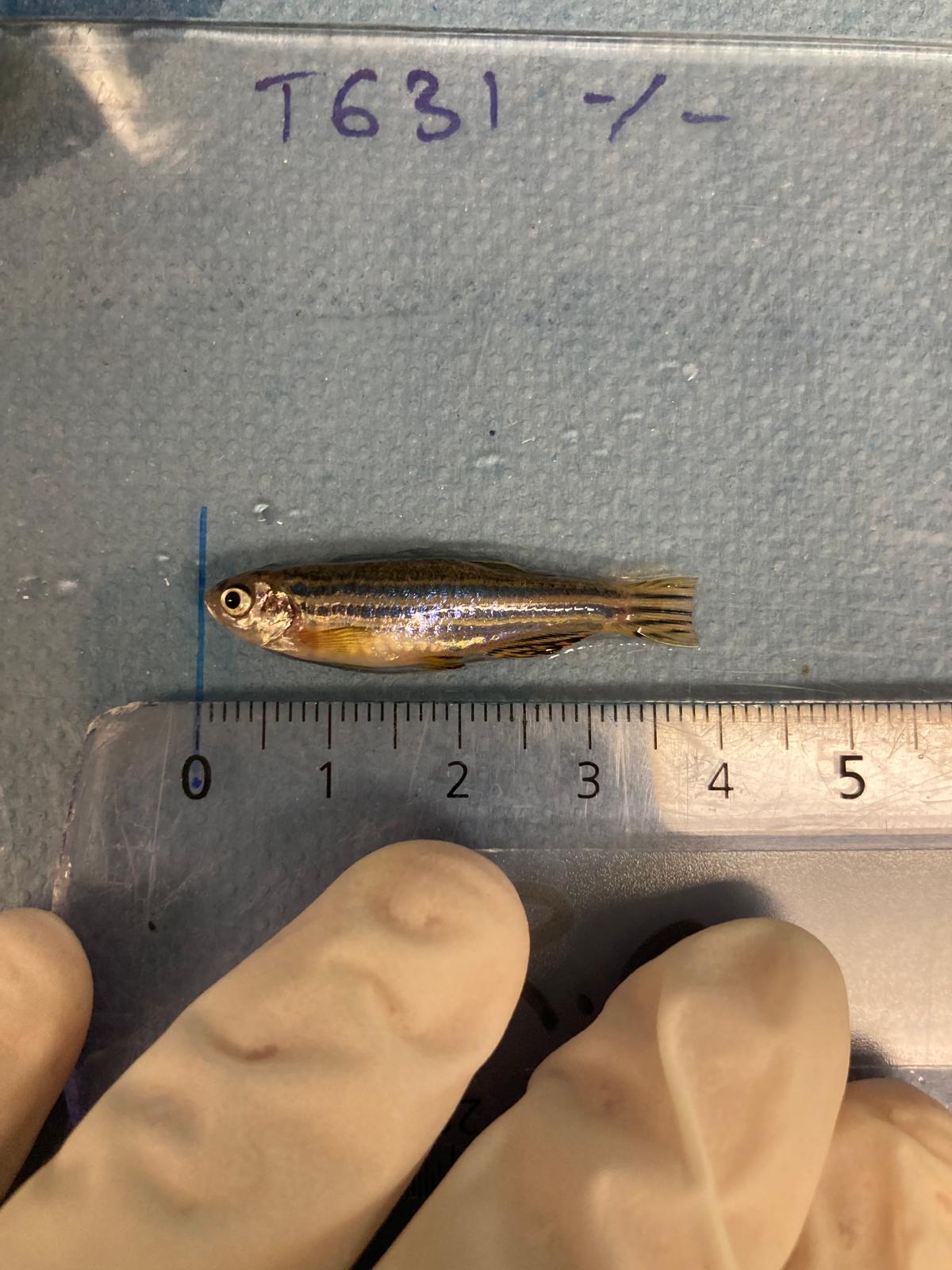

Supplement: Supplementary file 21 — Appendix Figure Source Data [file 44318_2025_588_MOESM21_ESM.zip › Figure S4/S4A/Adult il26-:-.jpeg]

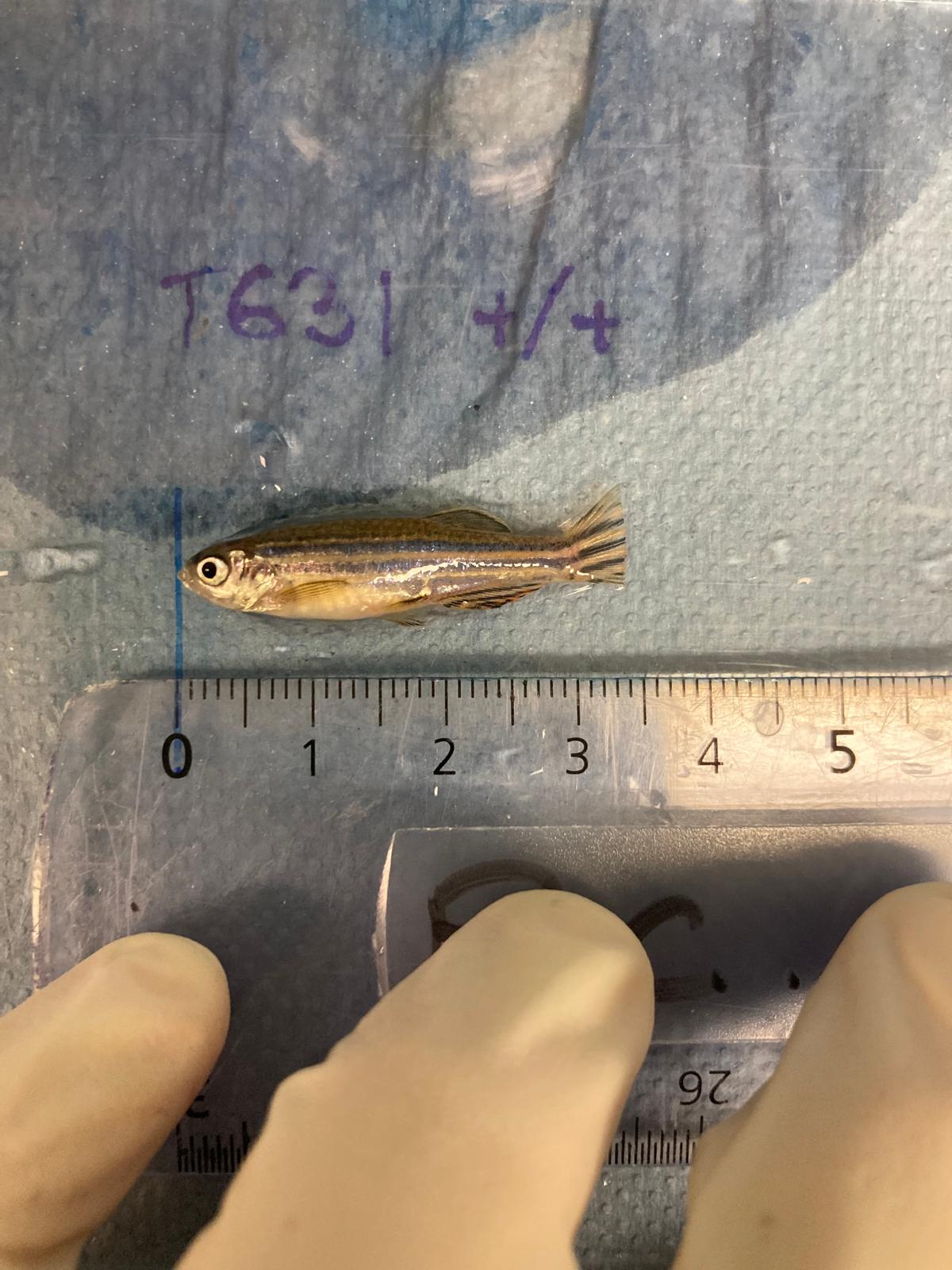

Supplement: Supplementary file 21 — Appendix Figure Source Data [file 44318_2025_588_MOESM21_ESM.zip › Figure S4/S4A/Adult il26+:+.jpeg]

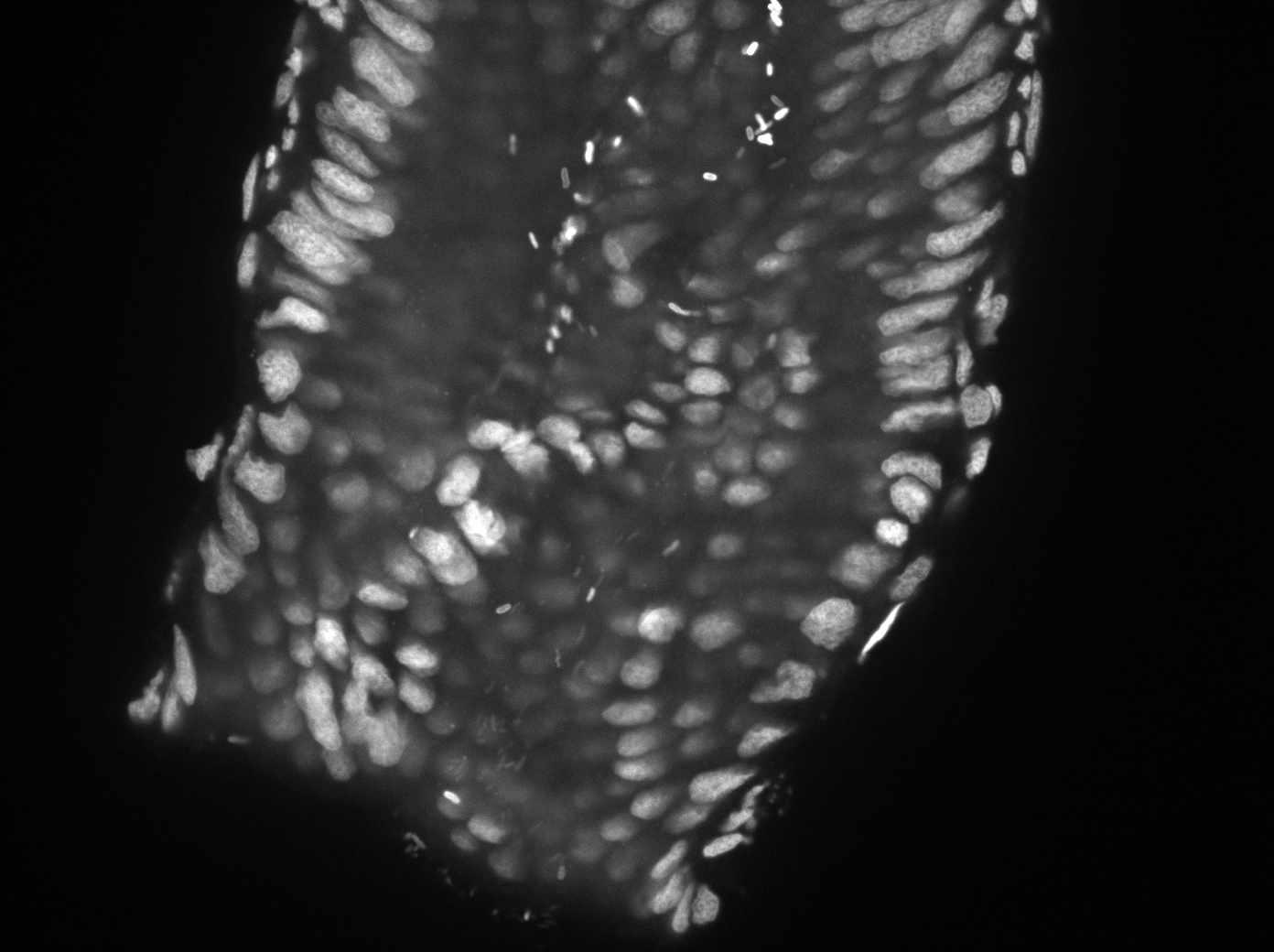

Supplement: Supplementary file 21 — Appendix Figure Source Data [file 44318_2025_588_MOESM21_ESM.zip › Figure S5/S5C/yH2AX and EdU staining in il20rako.tif]

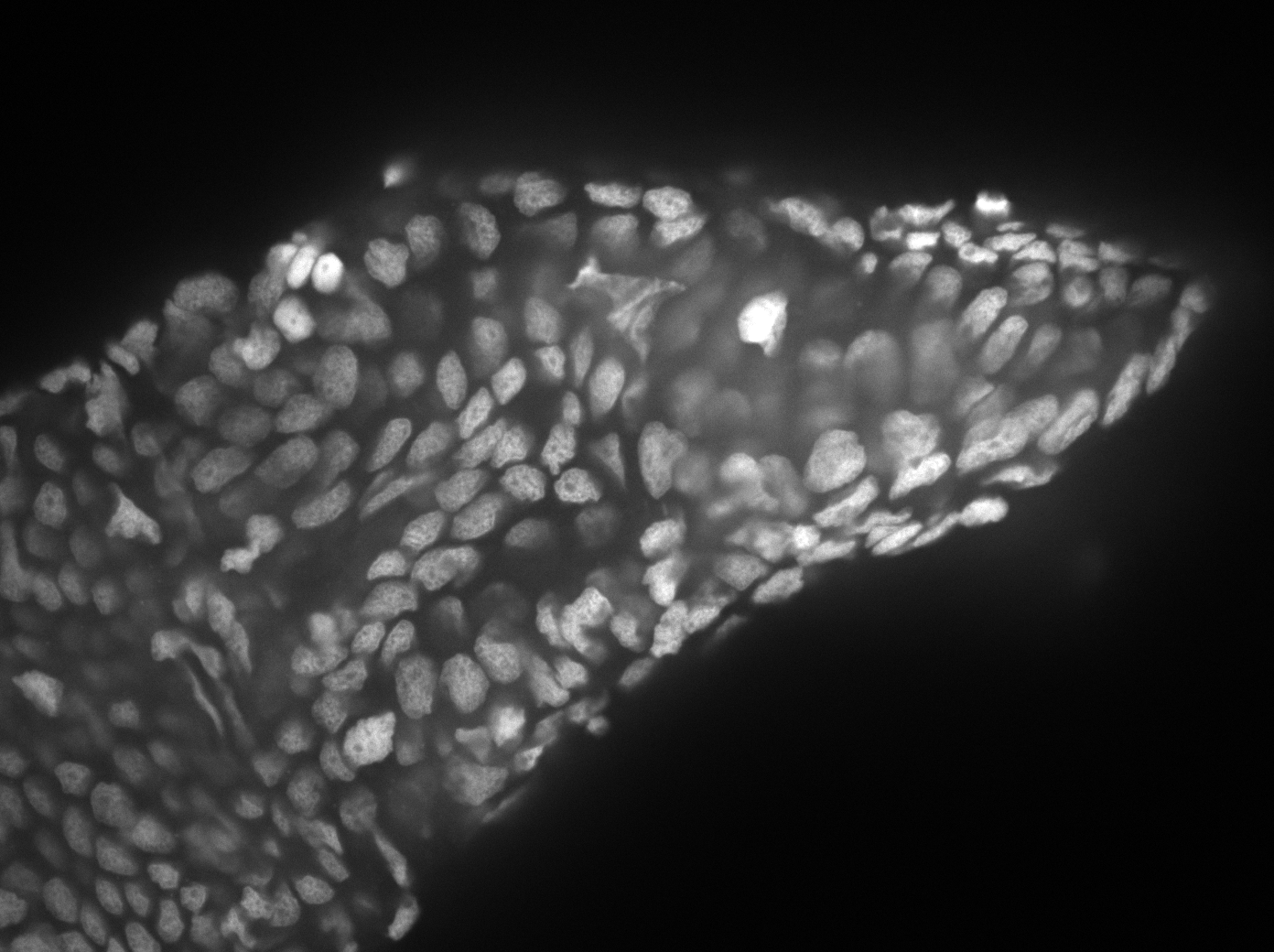

Supplement: Supplementary file 21 — Appendix Figure Source Data [file 44318_2025_588_MOESM21_ESM.zip › Figure S5/S5C/yH2AX and EdU staining in WT.tif]

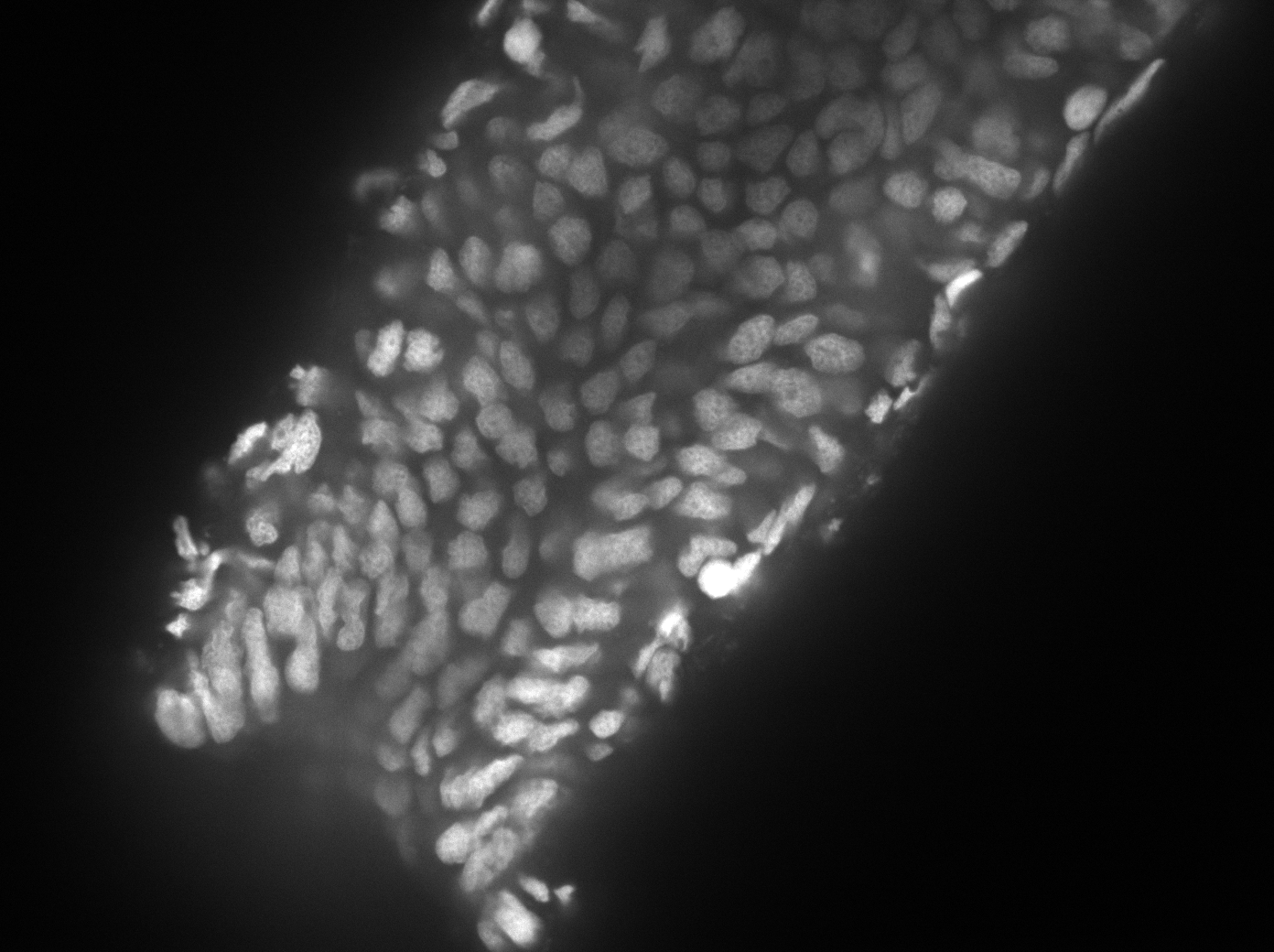

Supplement: Supplementary file 21 — Appendix Figure Source Data [file 44318_2025_588_MOESM21_ESM.zip › Figure S6/S6A/EdU in il26ko cohoused.tif]

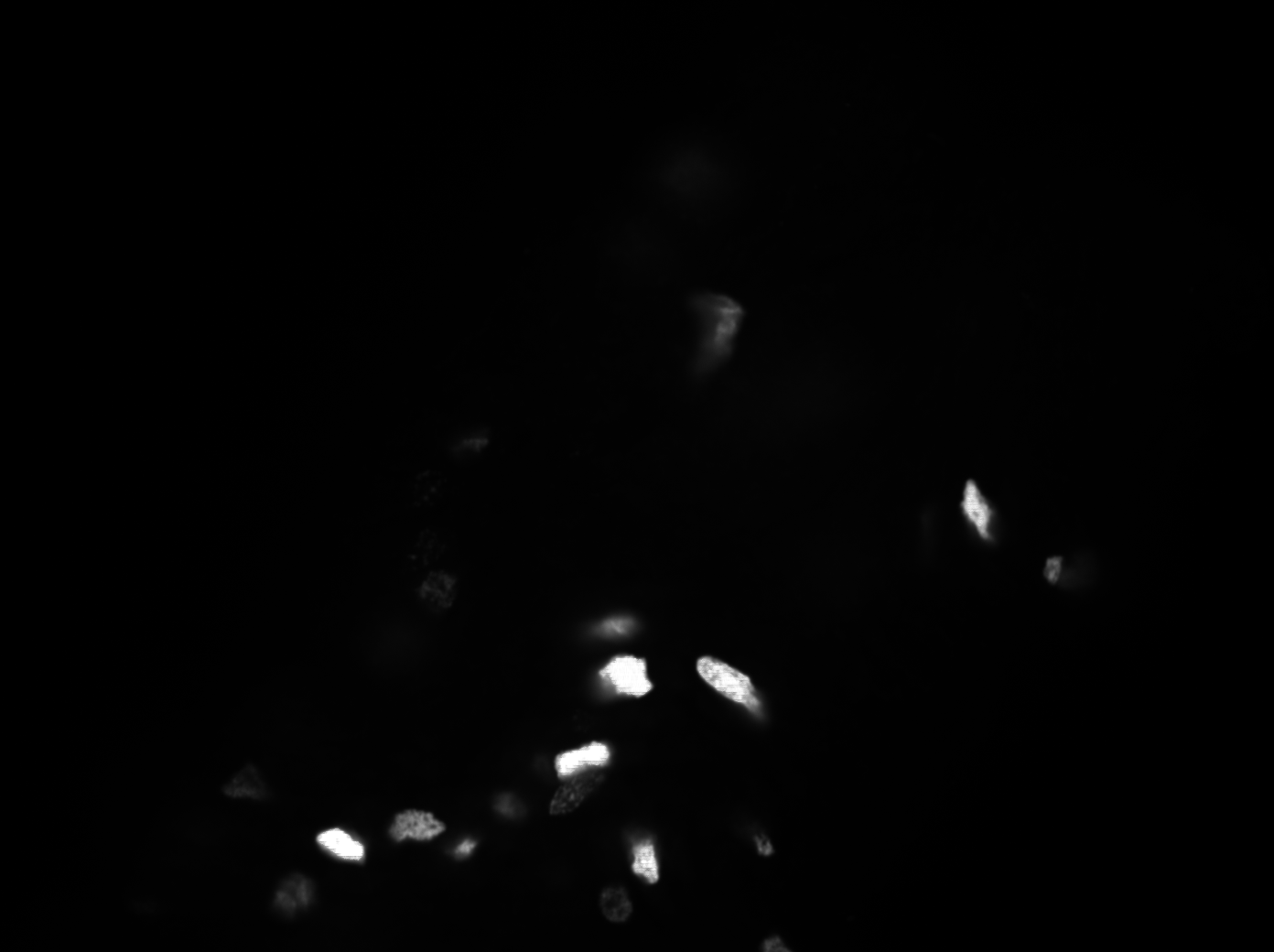

Supplement: Supplementary file 21 — Appendix Figure Source Data [file 44318_2025_588_MOESM21_ESM.zip › Figure S6/S6A/EdU in WT conventional.tif]

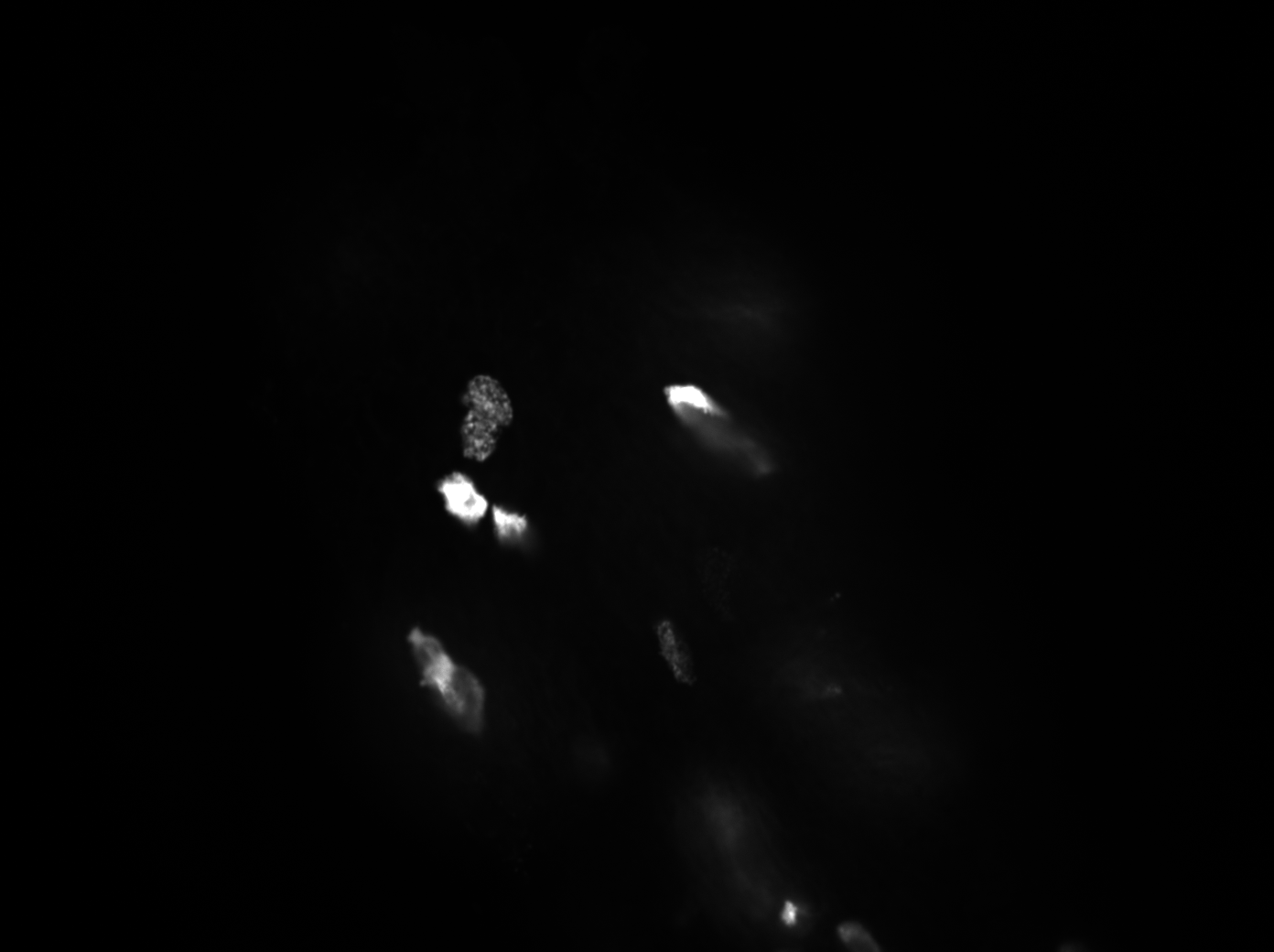

Supplement: Supplementary file 21 — Appendix Figure Source Data [file 44318_2025_588_MOESM21_ESM.zip › Figure S6/S6A/EdU in il26ko germ free.tif]

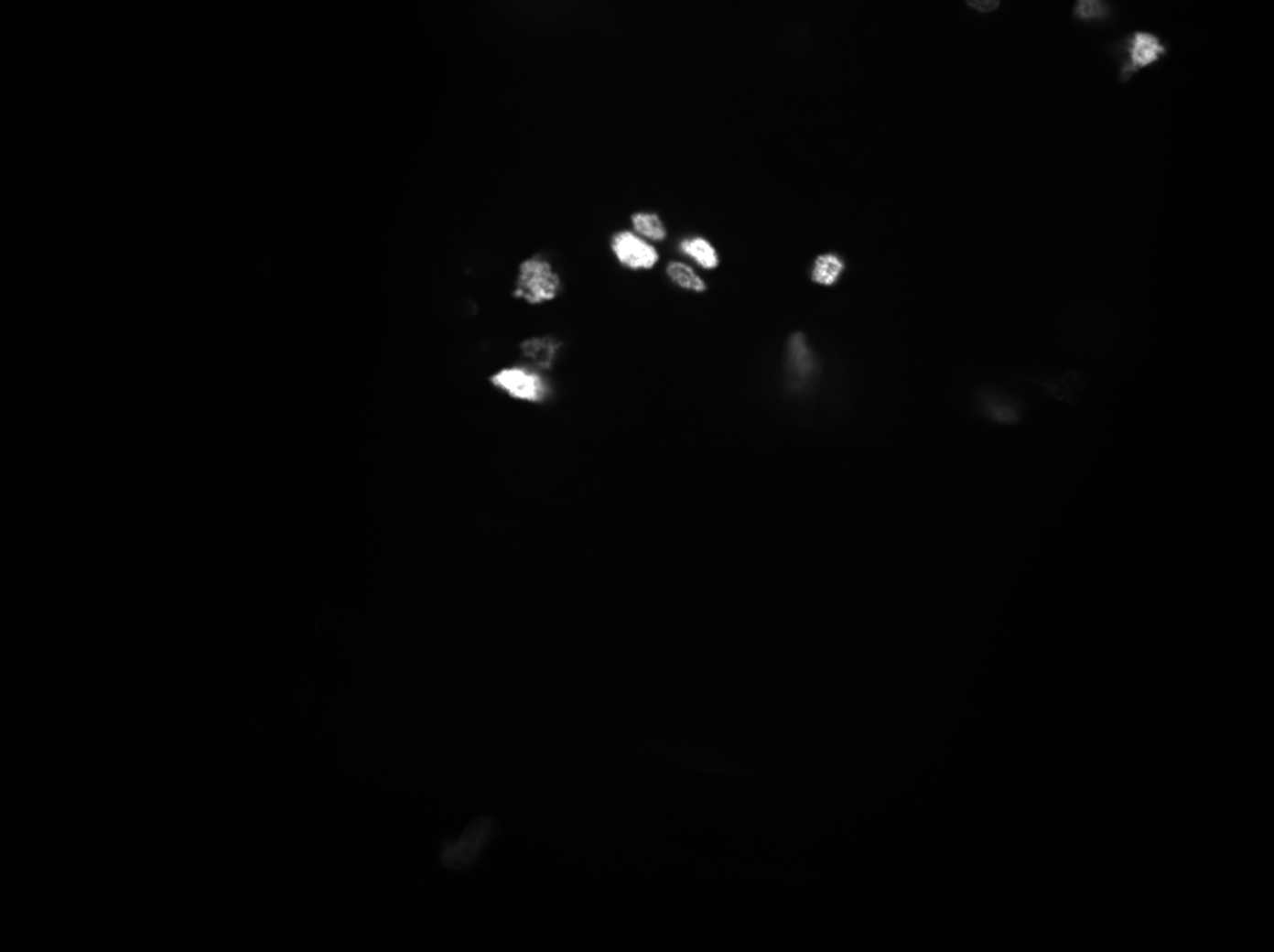

Supplement: Supplementary file 21 — Appendix Figure Source Data [file 44318_2025_588_MOESM21_ESM.zip › Figure S6/S6A/EdU in WT germ free.tif]

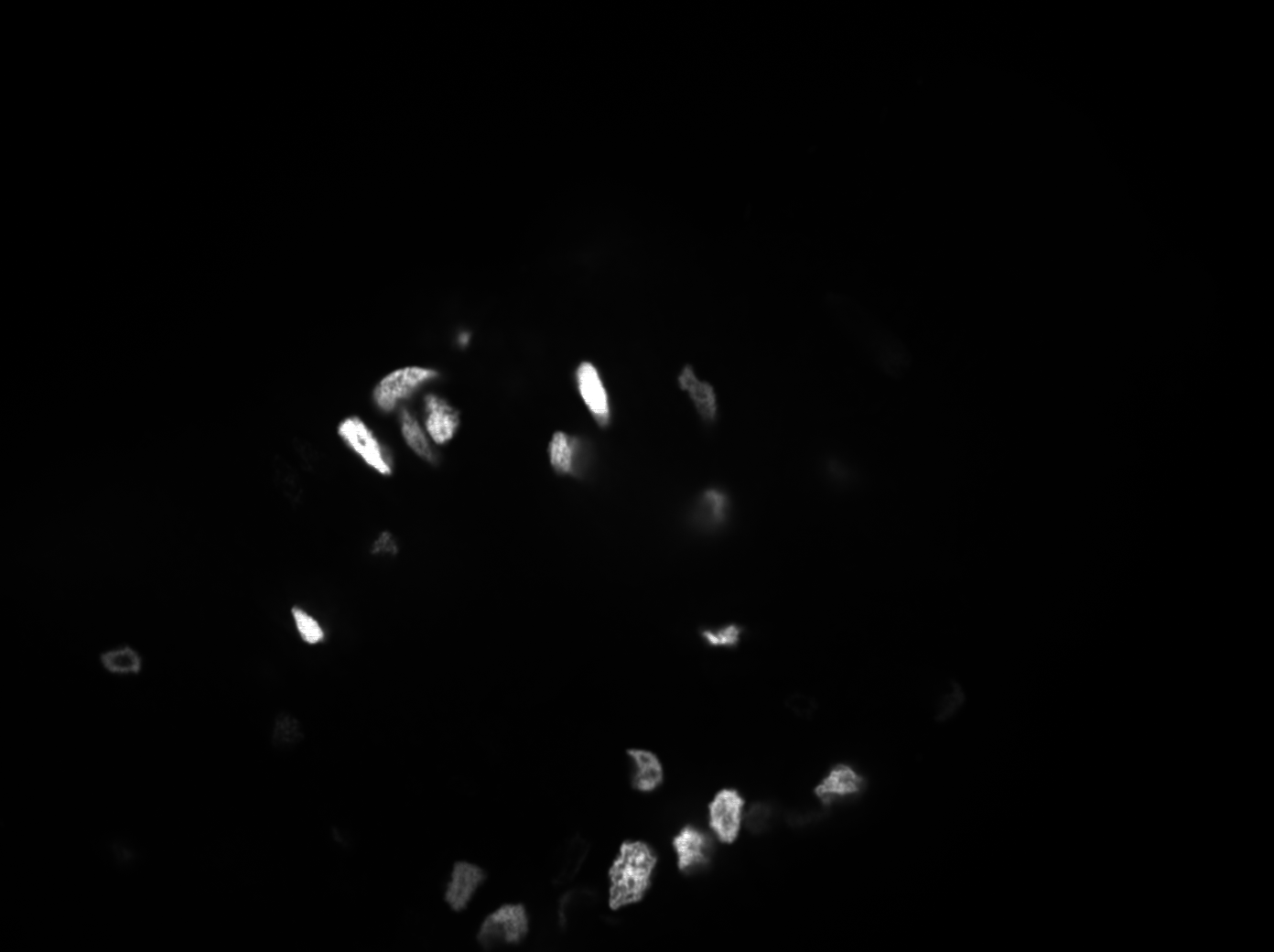

Supplement: Supplementary file 21 — Appendix Figure Source Data [file 44318_2025_588_MOESM21_ESM.zip › Figure S6/S6A/EdU in il26ko conventional.tif]

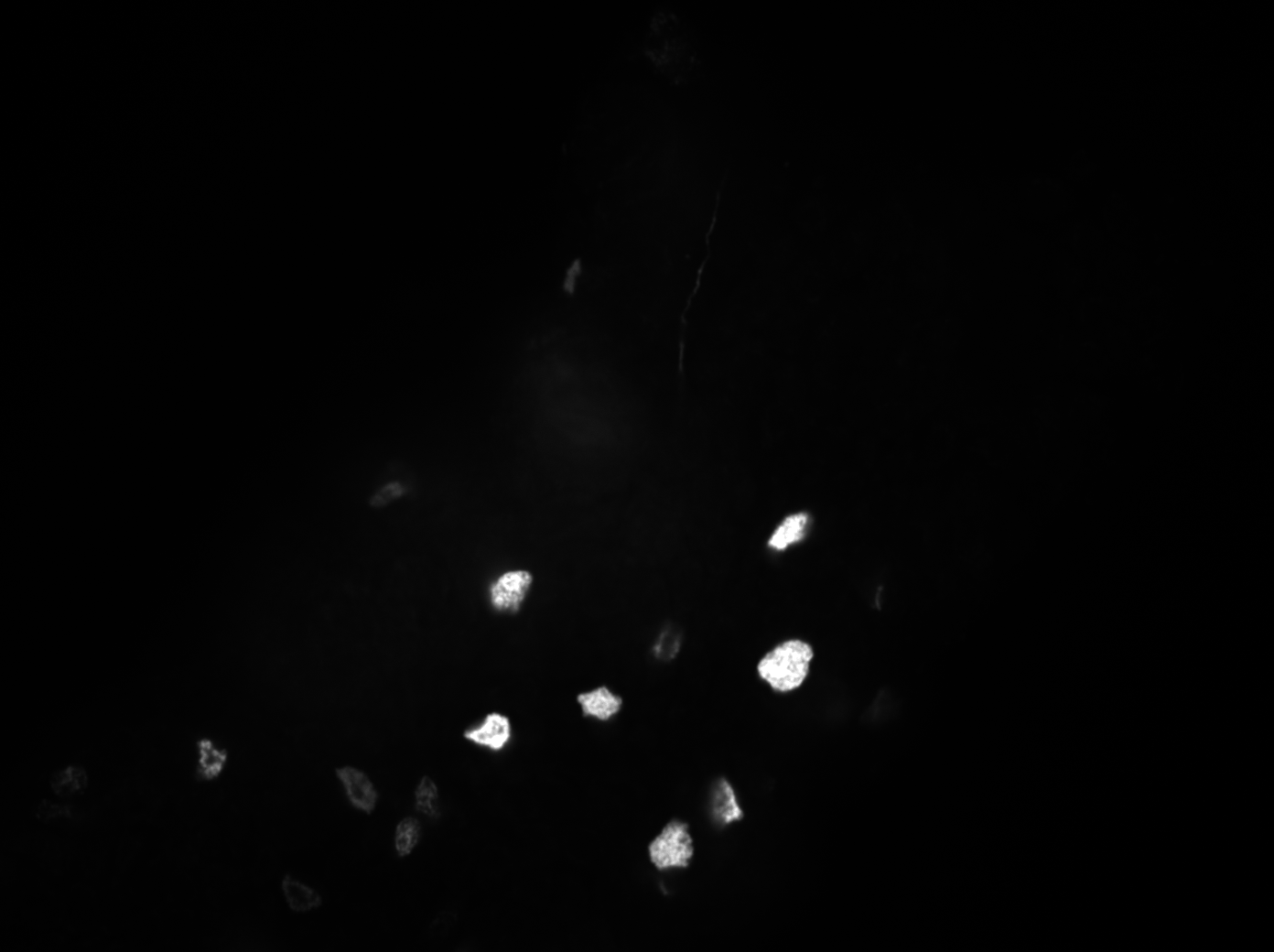

Supplement: Supplementary file 21 — Appendix Figure Source Data [file 44318_2025_588_MOESM21_ESM.zip › Figure S6/S6A/EdU in WT cohoused.tif]

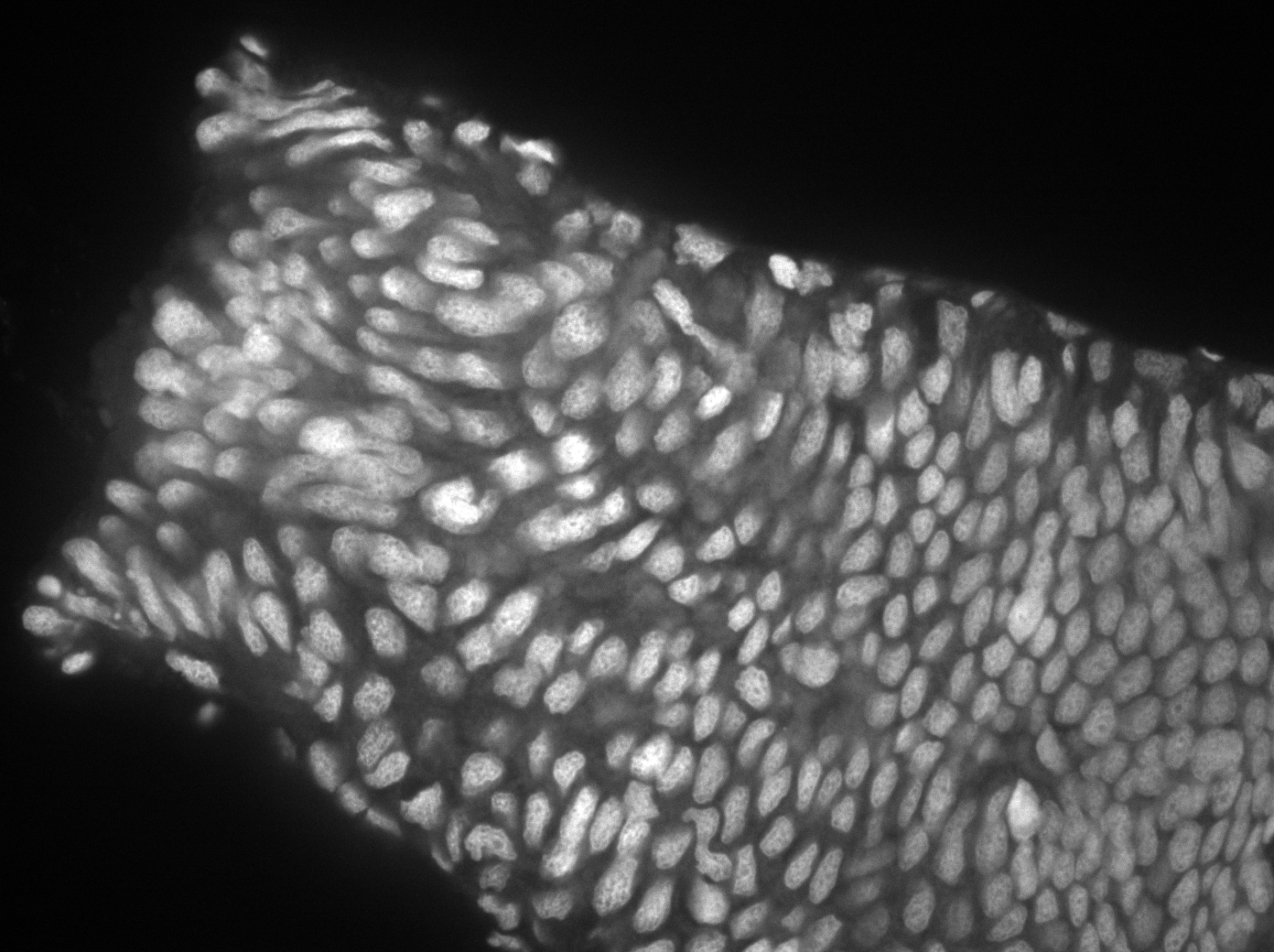

Supplement: Supplementary file 21 — Appendix Figure Source Data [file 44318_2025_588_MOESM21_ESM.zip › Figure S6/S6B/yH2AX in WT cohoused.tif]

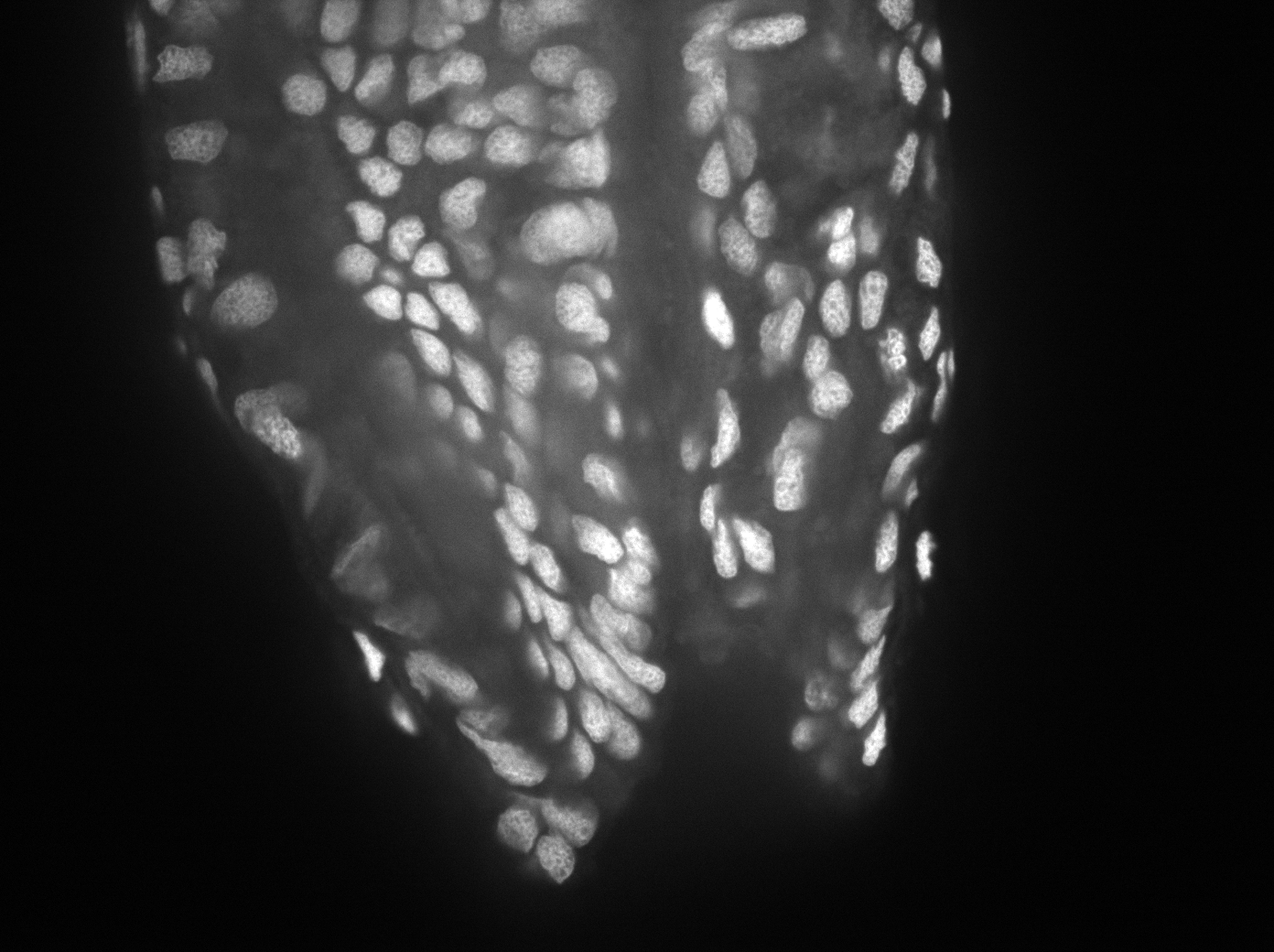

Supplement: Supplementary file 21 — Appendix Figure Source Data [file 44318_2025_588_MOESM21_ESM.zip › Figure S6/S6B/yH2AX in il26ko conventional.tif]

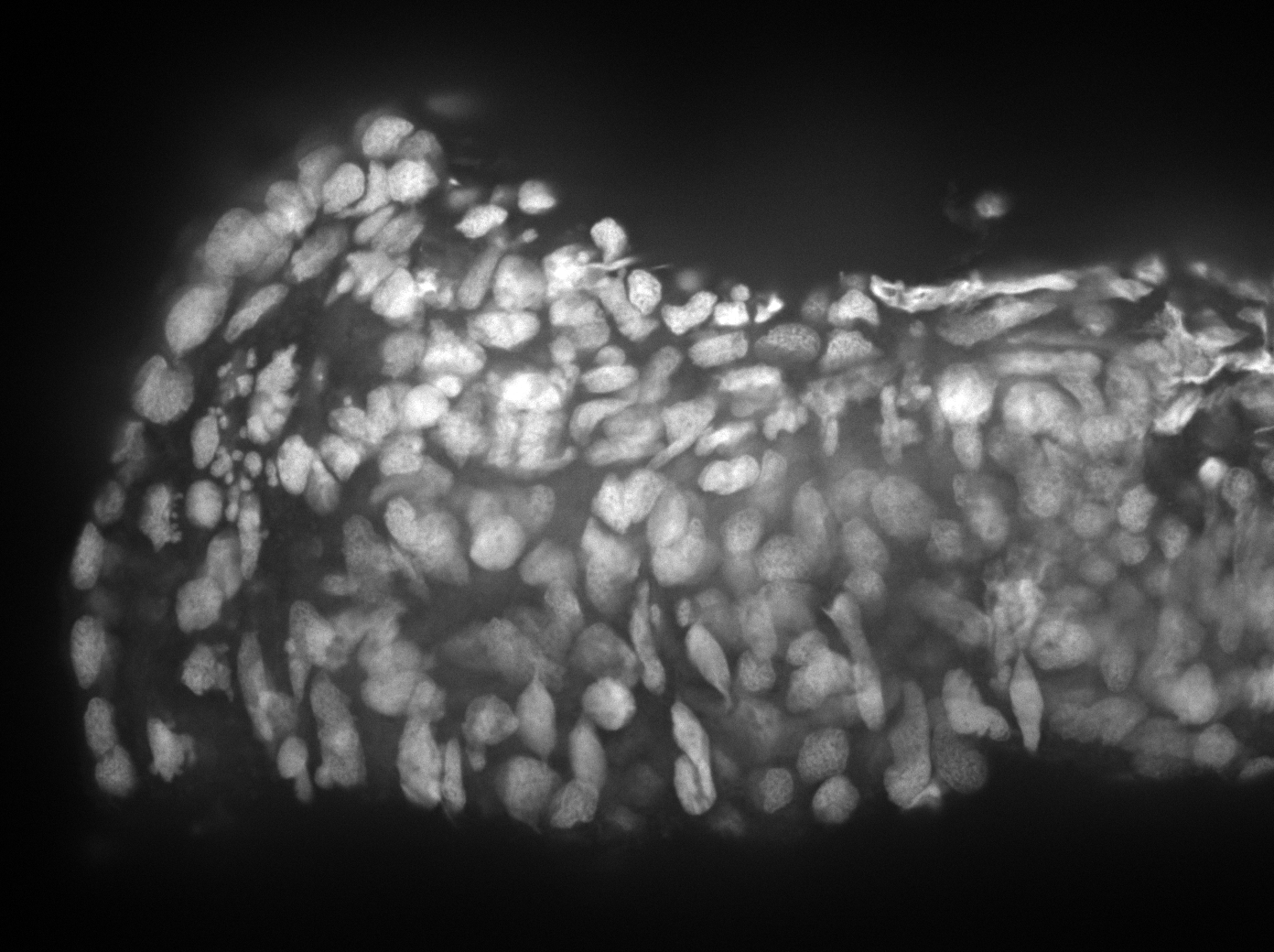

Supplement: Supplementary file 21 — Appendix Figure Source Data [file 44318_2025_588_MOESM21_ESM.zip › Figure S6/S6B/yH2AX in il26ko cohoused.tif]

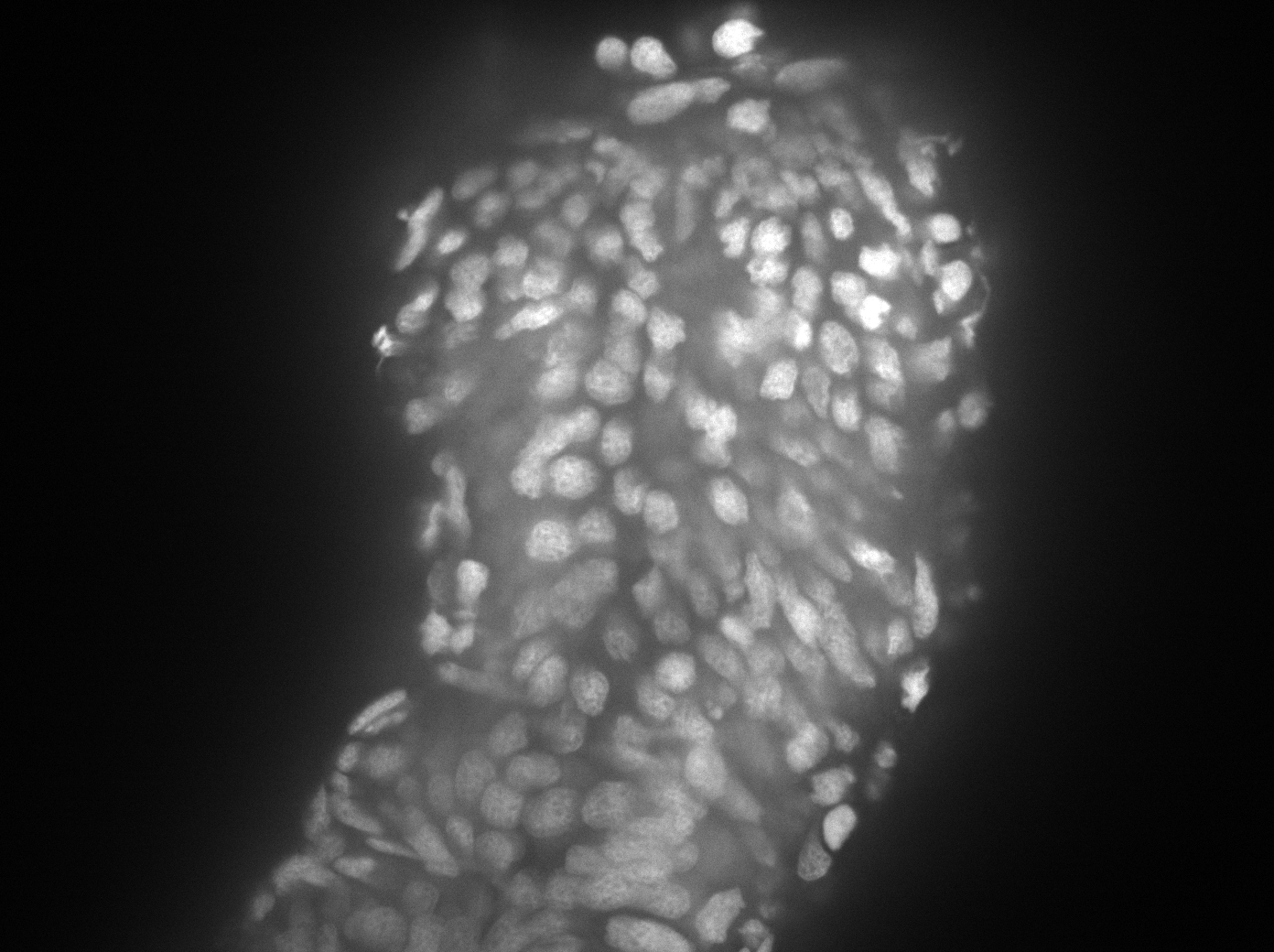

Supplement: Supplementary file 21 — Appendix Figure Source Data [file 44318_2025_588_MOESM21_ESM.zip › Figure S6/S6B/yH2AX in il26ko germ free.tif]

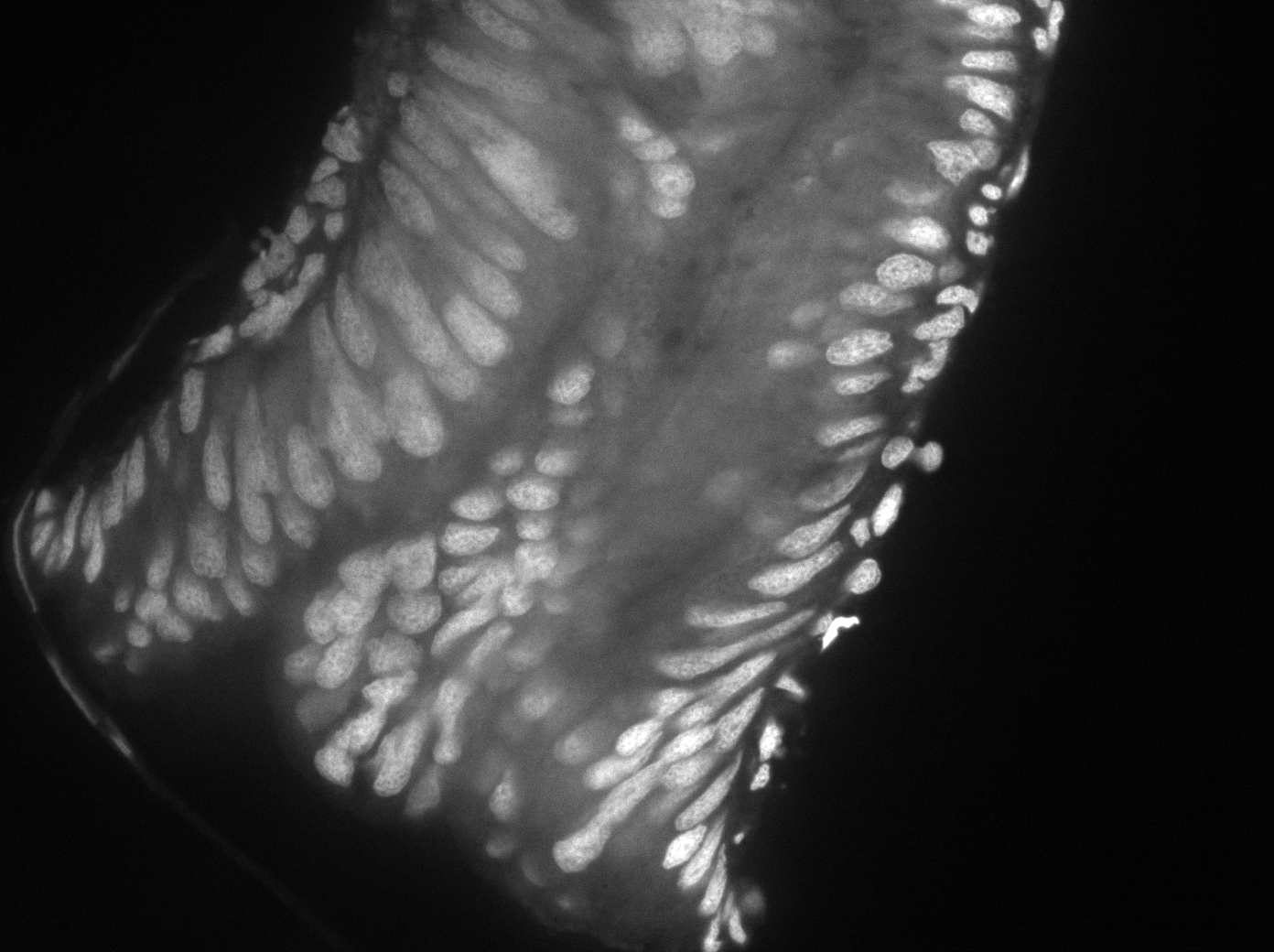

Supplement: Supplementary file 21 — Appendix Figure Source Data [file 44318_2025_588_MOESM21_ESM.zip › Figure S6/S6B/yH2AX in WT conventional.tif]

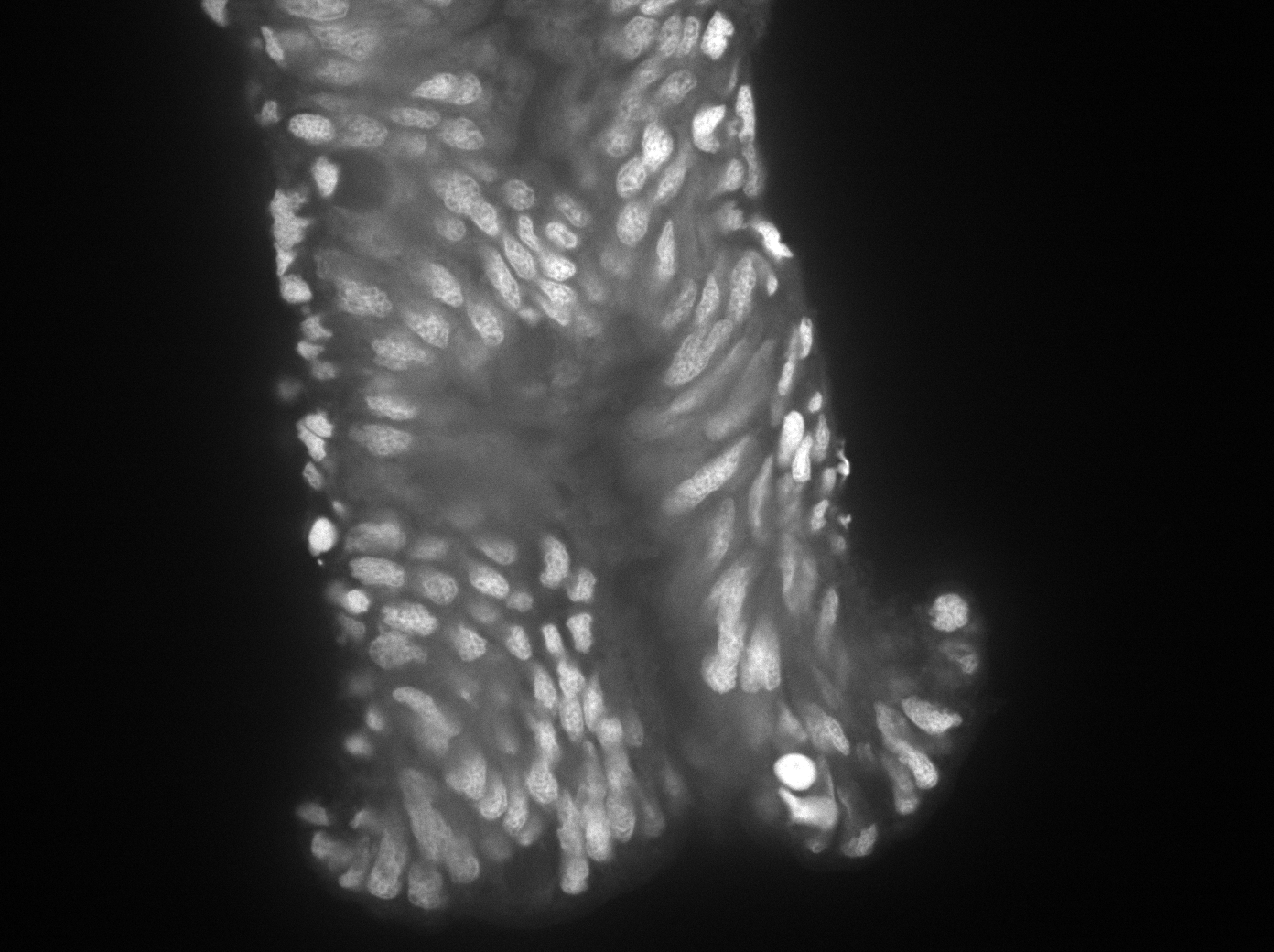

Supplement: Supplementary file 21 — Appendix Figure Source Data [file 44318_2025_588_MOESM21_ESM.zip › Figure S6/S6B/yH2AX in WT germ free.tif]

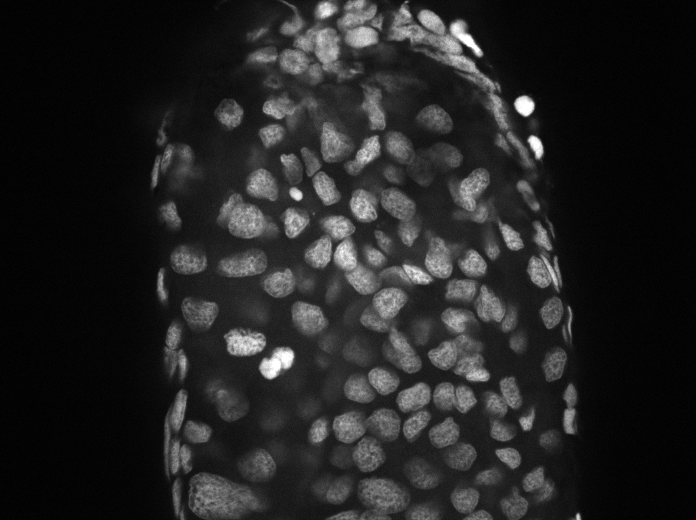

Supplement: Supplementary file 21 — Appendix Figure Source Data [file 44318_2025_588_MOESM21_ESM.zip › Figure S7/S7/il26ko uninfected.tif]

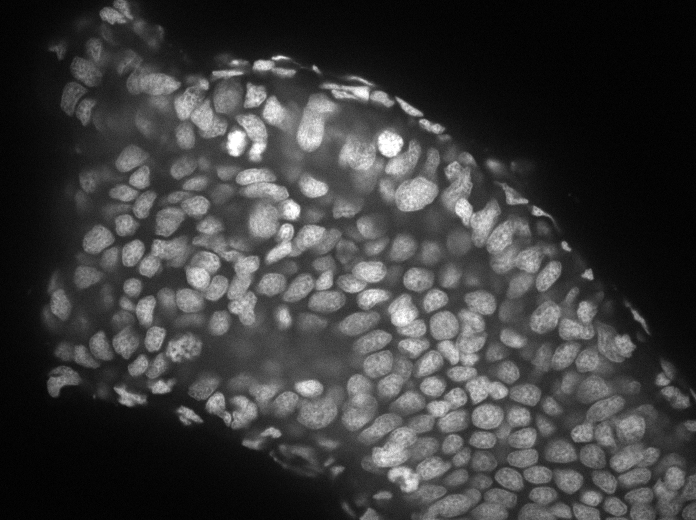

Supplement: Supplementary file 21 — Appendix Figure Source Data [file 44318_2025_588_MOESM21_ESM.zip › Figure S7/S7/il26ko infected.tif]

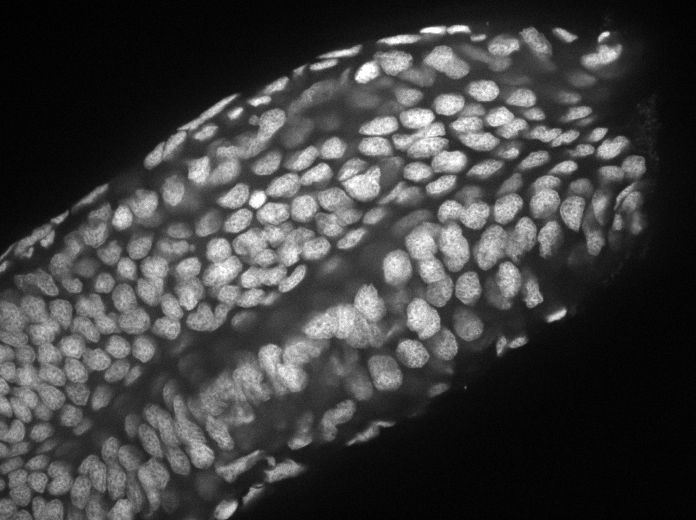

Supplement: Supplementary file 21 — Appendix Figure Source Data [file 44318_2025_588_MOESM21_ESM.zip › Figure S7/S7/WT infected.tif]

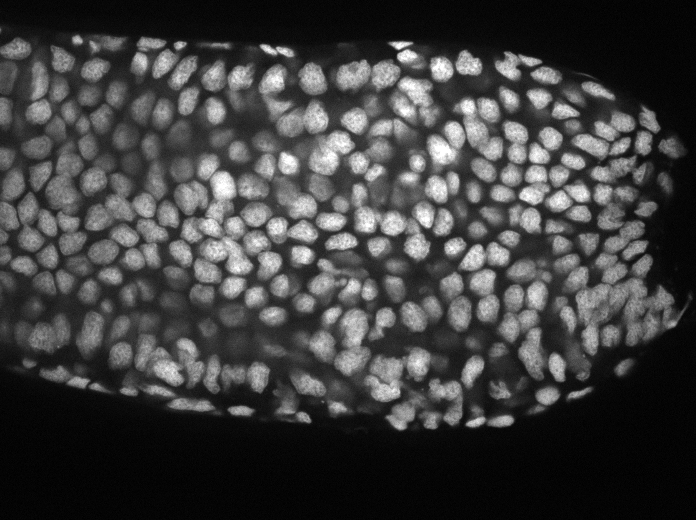

Supplement: Supplementary file 21 — Appendix Figure Source Data [file 44318_2025_588_MOESM21_ESM.zip › Figure S7/S7/WT uninfected.tif]
