# Supplementary material for: IL-26 from innate lymphoid cells regulates early-life gut epithelial homeostasis by shaping microbiota composition
Source: EMBO J. 2025 Oct 22;44(23):6832–56. doi: 10.1038/s44318-025-00588-w (PMC12669248; doi:10.1038/s44318-025-00588-w)
Supplement: Supplementary file 11 — Source data Fig. 3 [file 44318_2025_588_MOESM11_ESM.zip › Figure 3/3A/README.rtf]

1st channel: DAPI2nd channel: yH2AX3rd channel: cldn15a-GFP
