# Supplementary material for: IL-26 from innate lymphoid cells regulates early-life gut epithelial homeostasis by shaping microbiota composition
Source: EMBO J. 2025 Oct 22;44(23):6832–56. doi: 10.1038/s44318-025-00588-w (PMC12669248; doi:10.1038/s44318-025-00588-w)
Supplement: Supplementary file 18 — Figure EV3 Source Data [file 44318_2025_588_MOESM18_ESM.zip › Figure EV3/EV3A/README.rtf]

1st channel: cldn15a-GFP2nd channel: DAPI3rd channel: EdU
