# Supplementary material for: IL-26 from innate lymphoid cells regulates early-life gut epithelial homeostasis by shaping microbiota composition
Source: EMBO J. 2025 Oct 22;44(23):6832–56. doi: 10.1038/s44318-025-00588-w (PMC12669248; doi:10.1038/s44318-025-00588-w)
Supplement: Supplementary file 22 — Expanded View Figures [file 44318_2025_588_MOESM22_ESM.pdf]

Expanded View Figures

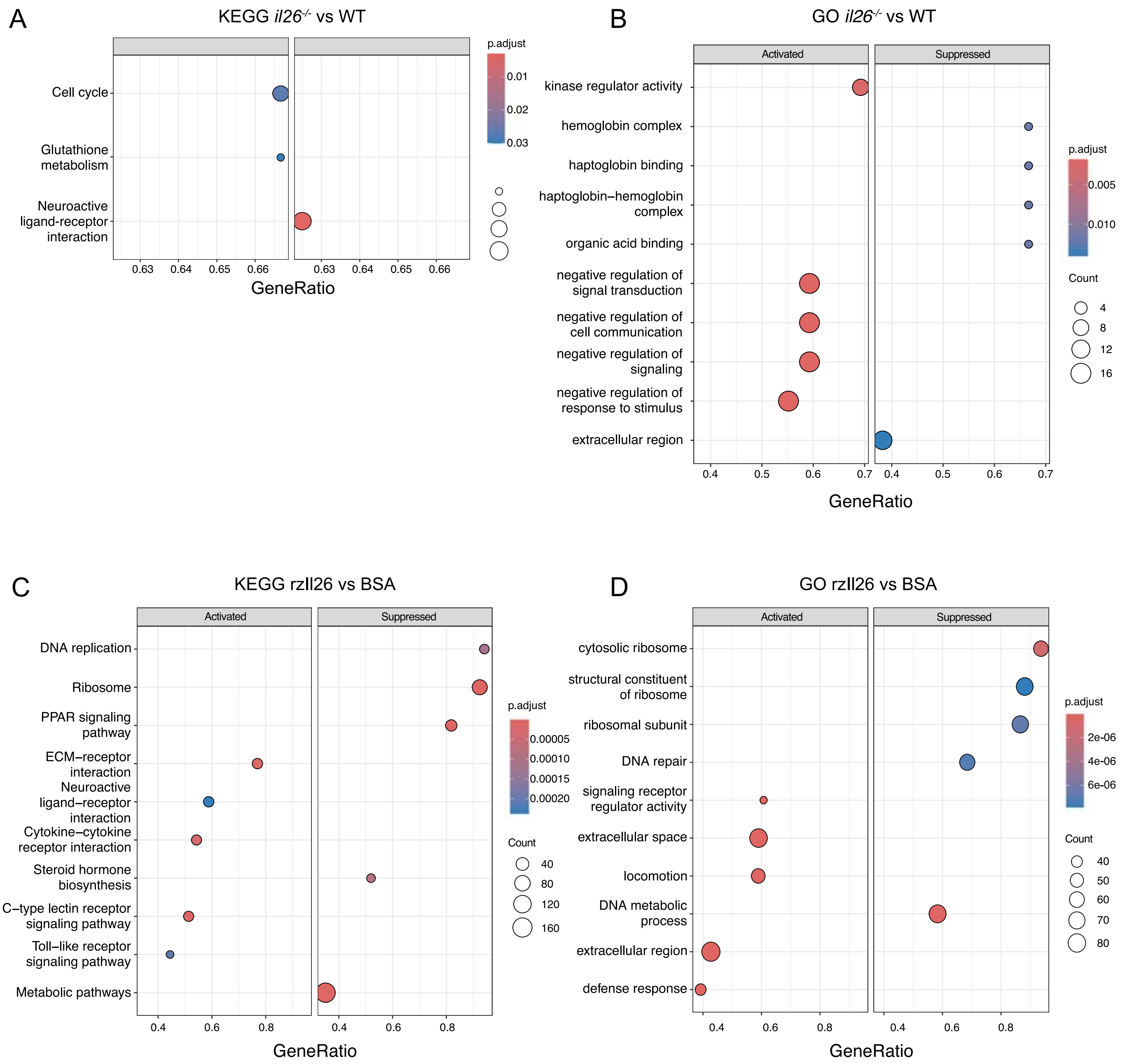

Figure EV1. Gene set enrichment analyses in IL-26 loss-of-function and overexpression datasets.

(**A, B**) KEGG pathway (**A**) and GO analysis (**B**) in the loss-of-function dataset. (**C, D**) KEGG pathway (**C**) and GO analysis (**D**) in the overexpression dataset. Statistical significance was determined by Kolmogorov-Smirnov-like permutation test using the ClusterProfile package in R.

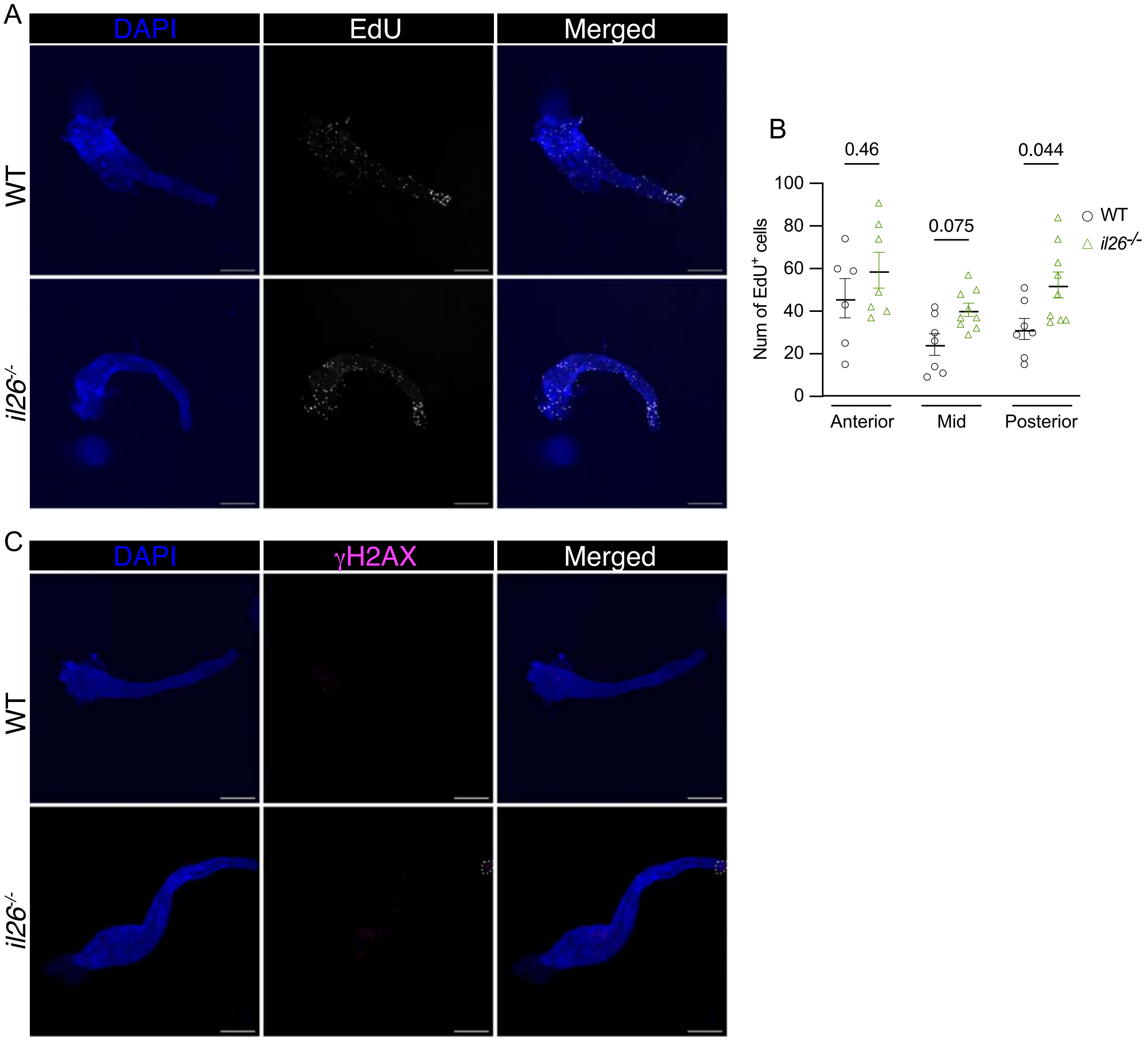

Figure EV2. IL-26 loss increases proliferation in the mid and posterior gut and DNA damage in the posterior gut in zebrafish larvae.

(**A**) Representative images of EdU staining in WT and *il26*$^{-/-}$ 5-dpf larval guts. Scale bars: 200 µm. (**B**) Quantification of EdU staining in gut segments of WT and *il26*$^{-/-}$ 5-dpf larvae. (**C**) Representative images of γH2AX staining in WT and *il26*$^{-/-}$ 5-dpf larval guts. Scale bars: 200 µm. Data information: (**B**) Data are presented as mean ± SEM. Sample sizes were as follows ((**B**): $n_{WT} = 7$, $n_{il26-/-} = 8$). n represents the number of biological replicates (minimum of 3 independent experiments). Statistical significance was determined by Mann–Whitney test (**B**). Source data are available online for this figure.

 © The Author(s)

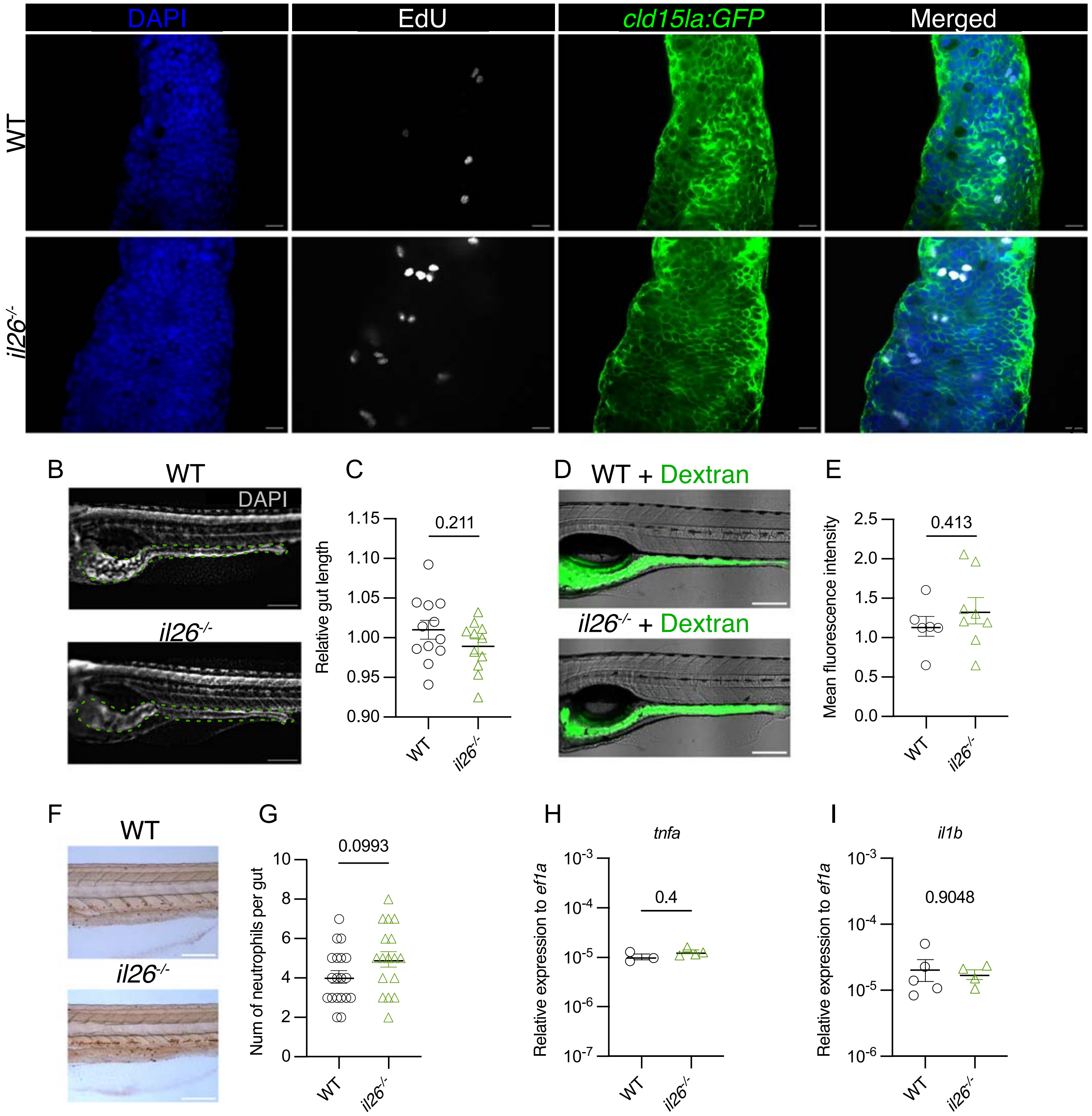

Figure EV3. Characterization of *il26-/-* zebrafish larvae.

(**A**) EdU staining of 5-dpf *TgBAC(cldn15la-GFP)* larvae in WT and *il26-/-* genetic backgrounds. Scale bars: 10 μm. (**B**, **C**) Images of WT and *il26-/-* larvae (**B**) and their gut length (**C**). Scale bars: 200 μm. (**D**, **E**) Representative images (**D**) of intestinal barrier integrity analysis in WT and *il26-/-* assessed by fluorescence intensity of orally gavaged FITC-dextran in the dorsal somites (**E**). Scale bars: 200 μm. (**F**, **G**) Representative images (**F**) and quantification (**G**) of neutrophil staining in WT and *il26-/-*. Scale bars: 100 μm. (**H**, **I**) qRT-PCR analysis of *tnfa* (**H**) and *il1b* (**I**) in dissected guts of WT or *il26-/-*. Data information: (**C**, **E**, **G**, **H**) Data are presented as mean ± SEM. Sample sizes were as follows ((**C**): $n_{WT} = 12$, $n_{il26-/-} = 11$; (**E**): $n_{WT} = 6$, $n_{il26-/-} = 8$; (**G**): $n_{WT} = 19$, $n_{il26-/-} = 18$; (**H**): $n_{WT} = 3$, $n_{il26-/-} = 4$; (**I**): $n_{WT} = 5$, $n_{il26-/-} = 4$). n represents the number of biological replicates (minimum of 3 independent experiments). Statistical significance was determined by Mann-Whitney test (**C**, **E**, **G**, **H**). Source data are available online for this figure.

© The Author(s)

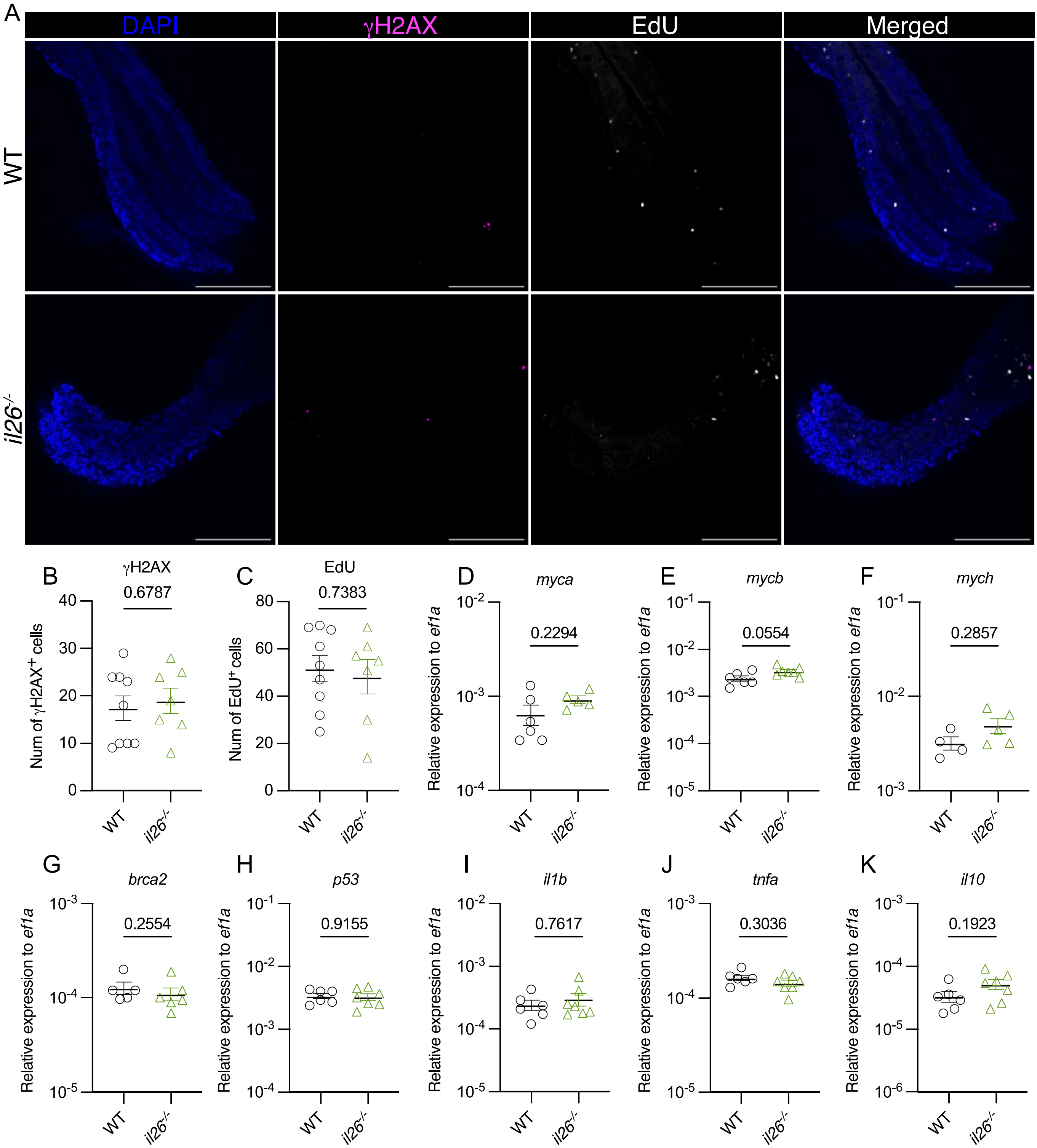

Figure EV4. Characterization of *il26*$^{-/-}$ juvenile zebrafish.

(**A**) Representative images of γH2AX and EdU staining in WT and *il26*$^{-/-}$ 5-wpf guts. Scale bars: 100 µm. (**B, C**) Quantification of γH2AX (**B**) and EdU staining (**C**) in WT and *il26*$^{-/-}$ 5-wpf posterior guts. (**D–K**) qRT-PCR analysis of selected genes in dissected guts of WT or *il26*$^{-/-}$ 5-wpf fish. Data information: (**B–K**) Data are presented as mean ± SEM. Sample sizes were as follows ((**B, C**): $n_{WT} = 9$, $n_{il26-/-} = 7$; (**D–K**): $n_{WT} = 4–6$, $n_{il26-/-} = 5–7$). n represents the number of biological replicates (minimum of 3 independent experiments). Statistical significance was determined by Mann–Whitney test (**B–K**). Source data are available online for this figure.

© The Author(s)

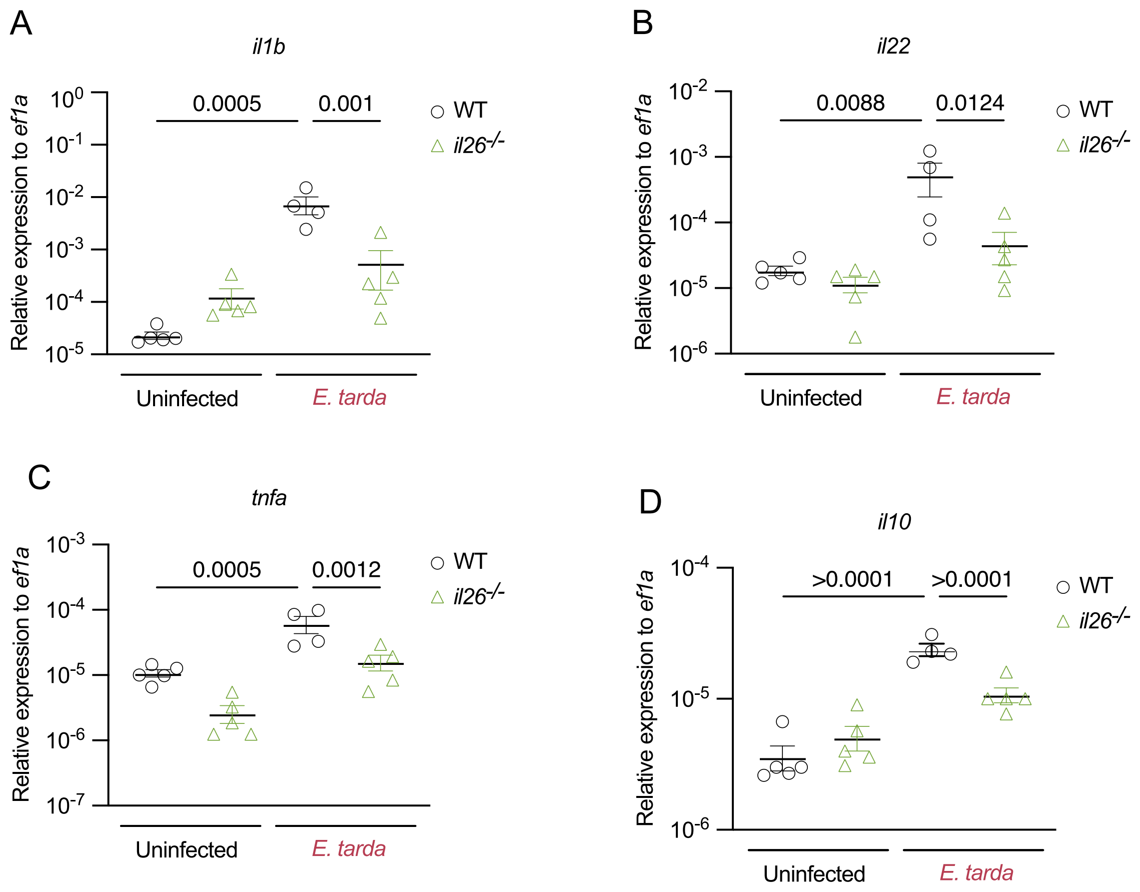

Figure EV5. Cytokine expression in WT and *il26*$^{-/-}$ larval guts following *E. tarda* infection.

(**A**, **D**) qRT-PCR analysis of *il1b* (**A**), *il22* (**B**), *tnfa* (**C**), *il10* (**D**) in dissected guts of WT and *il26*$^{-/-}$ at 3 dpi. Data information: (**A–D**) Data are presented as mean ± SEM. Sample sizes were as follows ((**A–D**): $n_{WT\ uninfected} = 5$, $n_{WT\ infected} = 4$, $n_{il26-/-\ uninfected} = 5$, $n_{il26-/-\ infected} = 5$). n represents the number of biological replicates (minimum of 3 independent experiments). Statistical significance was determined by one-way ANOVA (**A–D**). Source data are available online for this figure.

© The Author(s)
